# Lithospheric structural dynamics and geothermal modeling of the Western Arabian Shield

**DOI:** 10.1038/s41598-023-38321-4

**Published:** 2023-07-20

**Authors:** Oualid Melouah, Ebong D. Ebong, Kamal Abdelrahman, Ahmed M. Eldosouky

**Affiliations:** 1 Earth and Space Sciences Department, Faculty of Hydrocarbons, Renewable Energy and Earth and Space Sciences, University Kasdi Merbah Ouargla, 30000 Ouargla, Algeria; 2 Applied Geophysics Programme, Department of Physics, University of Calabar, PMB 1115, Calabar, Cross River State Nigeria; 3 Department of Geology & Geophysics, College of Science, King Saud University, P.O. Box 2455, Riyadh, 11451 Saudi Arabia; 4 Geology Department, Faculty of Science, Suez University, Suez, 43518 Egypt; 5 Academy of Scientific Research & Technology, Cairo, Egypt

**Keywords:** Solid Earth sciences, Geophysics

## Abstract

Understanding the dynamics of suturing and cratonisation and their implications are vital in estimating the link between the lithospheric mantle architecture and geothermal resources. We propose new interpretations of the Western Arabian Shield’s geodynamic styles and geothermal anomalies. In this work, features of the crust and mantle were interpreted from geophysical modeling to unravel the structural dynamics between the Arabian Shield and the Red Sea rift, as well as the influence of these mechanisms on the uplift of the Cenozoic basalts. Estimates of the lower crust thermal properties were also achieved. Spectral properties of the potential field were used to define the Curie isotherm, heat fluxes, geothermal gradients, radiogenic heat production, Moho configuration, and lithosphere-asthenosphere boundary. Results show new structural styles, micro-sutures, and significant thermal anomalies. The defined geothermal patterns were inferred to be due to localized initiation of tectonic and asthenospheric disequilibrium during the rifting episodes within the Red Sea. Also, magma mixing is initiated by the northward migration of magma from the Afar plume towards the Western Arabian Shield which drives local mantle melts beneath the western Arabia, thereby providing the pressure field required for magma ascent. The ascendant magma flow provides the heating source of geothermal reservoirs within the Western Arabian Shield. However, there are indications that during the episodes of rifting within the Red Sea and/or ancient Pan-African activities, the mixing process may have been altered resulting in crustal thinning and creating pathways of ascendant magma flow along the MMN volcanic line. Integrating geophysical and geothermal models indicated new zones of suturing and extensional tectonics between the amalgamated terranes. The geodynamic interpretation shows a new redistribution of terranes and continuous compressional and transtentional movements within the Arabian Shield.

## Introduction

The production of geothermal energy is primarily influenced by the depth of geothermal reservoirs^1^ and subsurface structural characteristics^2^. Geothermal resources with an observed temperature greater than 179 °C have been observed to be associated with locations that are in proximity to tectonic plate boundaries^1,3^. Such hydrothermal anomalies are recorded within the Western Arabian Peninsula resulting from oceanic floor spreading^4^. One major clean source of energy is geothermal energy and its characteristic advantage over other sources such as fossil fuel necessitated the Saudi Arabian Authority to include the development of geothermal energy in its energy diversification program. Studies show that geothermal energy plants produce minimum CO_2_ emissions, i.e., ~ 0.893 kg of CO_2_ per megawatt hour, while fossil fuel energy plants emit ~ 193 to 817 kg of CO_2_ per megawatt hour^1^. However, the origin of geothermal anomalies is not just significant for energy production and economic processes, but also facilitates the interpretation of major geodynamic-structural characteristics of an area. Furthermore, it is imperative to understand the origin and processes that lead to geothermal reservoir formation, as this is a vital component of geothermal resource potential evaluation and development^2,5,6^.

Spectral properties of aeromagnetic data are critical for evaluating the lithospheric deformations and deep crustal behaviors can be assessed based on the estimation of Curie isotherm. At temperatures greater than 580 °C, rocks generally assume nonmagnetic conditions, providing a means of evaluating the Curie depth isotherm from spectral techniques^7,8^. Several factors such as the Moho depth, lithosphere-asthenosphere thermal disequilibrium, and radioactive heat production emanating from the lithospheric mantle, have been observed to influence the distribution of the Curie depth isotherm and provide the key for interpreting the origin of geothermal anomalies and understanding its genetic mechanism on sedimentary basins^9–11^.

In Saudi Arabia, there is a knowledge gap with regard to the potentials of geothermal resources^12^, hence, the need for this comprehensive research; to close the geothermal resource knowledge gap and classify its genesis in relation to geodynamic activities within the Western Arabian Shield. Major geothermal potentials are located in the southwestern Coastline and in the Harrats (Cenozoic basalt), the proximity of the potential zones of heat production from urban areas is an important consideration when developing geothermal resources for energy generation and water desalination as this can reduce considerably, costs and efforts for energy transfer. Studies on geothermal energy started with the works of^13^ which focused on the hydro-chemical properties of Jizan and Al-Lith hot springs. During the period (i.e., 1991–2006), improved the geologic knowledge regarding the petrography and petrochemical compositions of basaltic rocks within the Arabian Shield^14–19^. Recently, several authors have discussed the future of geothermal energy in Saudi Arabia using multidisciplinary approaches^1,20–22^. Geothermal resources can be categorized into three major classes: (a) low enthalpy resources, that generally represented by sedimentary aquifers and characterized by normal geothermal gradient, (b) median enthalpy resources, observed within the southwestern and western coastlines and are represented by hot spring thermal resources, and (c) high enthalpy resources associated with the lava field or the Harrats (i.e., resulting from a volcanic eruption and are mainly composed of basalt) represent this category of geothermal resources and extends across the coastline of the Red Sea within the western segment of Saudi Arabia^12^.

In this study, the aim is to obtain realistic models of temperature, density, and P- and S-wave distribution within the crust and uppermost mantle to construct a tectono-thermal conceptual model of Western Arabian Shield using geological and potential field information. The modeling process can provide better control of thermodynamic parameters, particularly for the Arabian-Nubian transitional zone where conspicuous asthenospheric plume exist. Since complex asthenospheric upwelling can generate temperature and density changes within the amalgamated terranes, development of thermal-geophysical model can adequately resolve the conditions for such changes in crustal rocks. Hence, preliminary results of previous dataset and our 2D model were combined to advance the knowledge of structural styles and provide new insights into the thermal evolution of the area.

### Regional geologic setting

**Figure 1:**
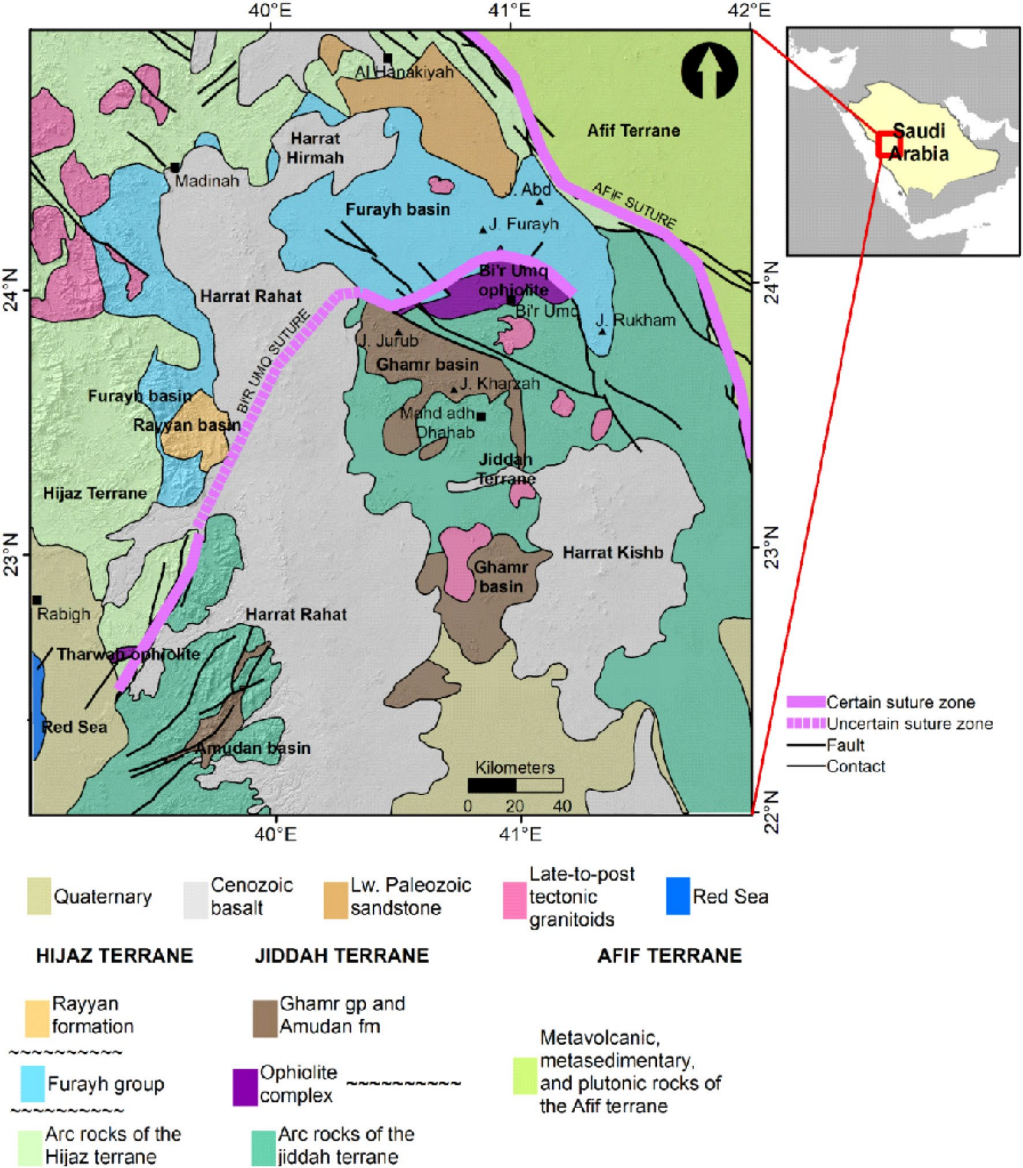
Geologic map showing the dominant structural elements and their limits within the western Arabian Shield (US Geological survey of America and the Arabian American Oil company, https://nla.gov.au/nla.obj-2458846831/view), the transects A–A′, B–B′, and C–C′ represents the thermal/seismic modeling profiles. Map generated using ArcGIS Pro (https://pro.arcgis.com/en/pro-app/latest/get-started/download-arcgis-pro.htm).

The Western Arabian Shield (WAS) covers an expanse of Precambrian rocks, mainly igneous, metamorphic, and belts of sedimentary rocks as well as young volcanics with varying degrees of sutures (Fig. 1). The opening of the Red Sea has immensely altered the WAS.

The present-day WAS is the result of several recent geologic events. In time past, preceding the aforementioned event (~ 30 Ma) the Arabian shield covers an area of ~ 650,000 km^2^ and partly constituted the Arabian-Nubian geologic province^21^. The Arabian-Nubian Shield (ANS) is a representation of a juvenile Neoproterozoic crustal accretion encapsulated in two distinct supercontinental cycles, i.e., the Rodinia breakup and the amalgamation of the Gondwana^23^. The ANS originated from the assemblage and accretion of Proterozoic island arc terranes (800–670 Ma). This episode occurred at some stage during the dismembering of the Rodinia supercontinent, i.e., between Tonian and early Cryogenian (870–800 Ma), within the Mozambique Ocean^24–26^. Terranes convergence and amalgamation around the Mozambique Ocean caused by intraoceanic arc–arc collision and eventually island arc collision with Saharan Metacraton^26^, led to high-strain shear zones, i.e., sutures marked by ophiolites and refolded recumbent folds^27,28^. However, some of these sutures were altered by strike-slip deformations^26^.

Tectonic developments during the Neoproterozoic in the Arabian shield can be categorized into; the oceanic, arc-accretion, and extensional phases. The oceanic phase, left relicts of dismembered ophiolites, currently found in tectonic mélanges. Localized ophiolites (870–740 Ma) sequences have been reported together with sub-parallel dykes, pillow-like lavas, gabbros, peridotites, and sedimentary rocks that showed evidence of deep-sea origin^29,30^. In the adjoining Nubian shield, remnants of the arc-accretion phase (900–700 Ma) are dominant in rocks such as basalts, andesites, gabbros, tonalites, and the Neoproterozoic metavolcanics^30–32^. The oldest remnant of the island-arcs (900–850 Ma) on the Arabian shield is often referred to as immature island arcs and is composed of tholeiitic magma series that includes tholeiitic andesites and basalts^33,34^. Relatively mature island arcs were also formed in this phase due to tholeiitic crustal thickening and decompression melting between 825 and 730 Ma^35^. Discontinuous curvilinear belts of ophiolitic suites, interpreted as sutures have been reported across the ANS^36^. The duration within which the suturing occurred was from ~ 900 to 550 Ma, during the closure of the Mozambique Ocean^21,29^. Additionally, this episode may have caused the formation of an oceanic plateau due to the upwelling of mantle plume and decompression melting^21^. Several tectonostratigraphic terranes were formed due to the accretion events at different directions (e.g., north and north-east) and were separated by either associated serpentinized ultramafic rocks along major suture zones or dominant northwest-trending fault structures^21^.

The northern, eastern, and southern flanks within the Arabian Shield are enclosed by thin Phanerozoic sedimentary sequence. These sedimentary successions are often truncated by volcanic rocks, traceable to episodes of metamorphism, deformation, and syntectonic intrusions. The deformational processes due to the amalgamation and suturing of arcs led to several regional uplifts and the development of retro arc foreland basins on the newly formed continental crust that are more than 40 km thick^21,37^. Sedimentation within these basins and volcanic intrusions occurred contemporaneously, resulting in volcano-sedimentary successions that overlie the forearc accretionary prism in some areas^37^. Some other basins within the region were formed along strike-slip fault structures during transtensional and transpressional extensions along releasing and constraining bends respectively. Others may have been formed during episodes of normal faulting occasioned by crustal extension and doming^37,38^.

## Materials and methods

### Magnetic and gravity data

The magnetic data employed to explore the geothermal potentials of the Western Saudi Arabia was extracted from the global Earth Magnetic Anomaly Grid (EMAG2)^39^, compiled from magnetic observations, ship-borne, and airborne data which is an updated version of the Earth magnetic grid model (supple. Fig. 1), i.e., EMAG3^40^. The EMAG2 version witnessed an improvement in resolution from 3 arc-min to 2 arc-min, while the altitude reduced from 5 to 4 km above the geoid. Additional compiled grids and track lines have been updated to enhance the data resolution (supple. Fig. 1). EMAG2 is available at http://geomag.org/models/EMAG2. Magnetic field information is dipolar, i.e., constitutes the magnetic field component of interest and other unknown factors of the magnetic source that are not relevant for investigations of this sort. To suppress the effects of this dipolar nature due to the unknown factors influencing the magnetic field data, reduction-to-the-magnetic pole (RTP) was performed. The inclination and declination applied during the RTP correction procedure were 35° and 3.52°, respectively. Upward continuation was performed on the magnetic data to 2 km to enhance structures of regional interest. The interactive radially averaged power spectrum enhancement procedure that involves direct and inverse Fourier transforms was applied to the raw data during the determination of magnetic anomaly fields. The residual magnetic anomaly was obtained, after subtracting the regional field component from the preprocessed magnetic data.

**Figure 2:**
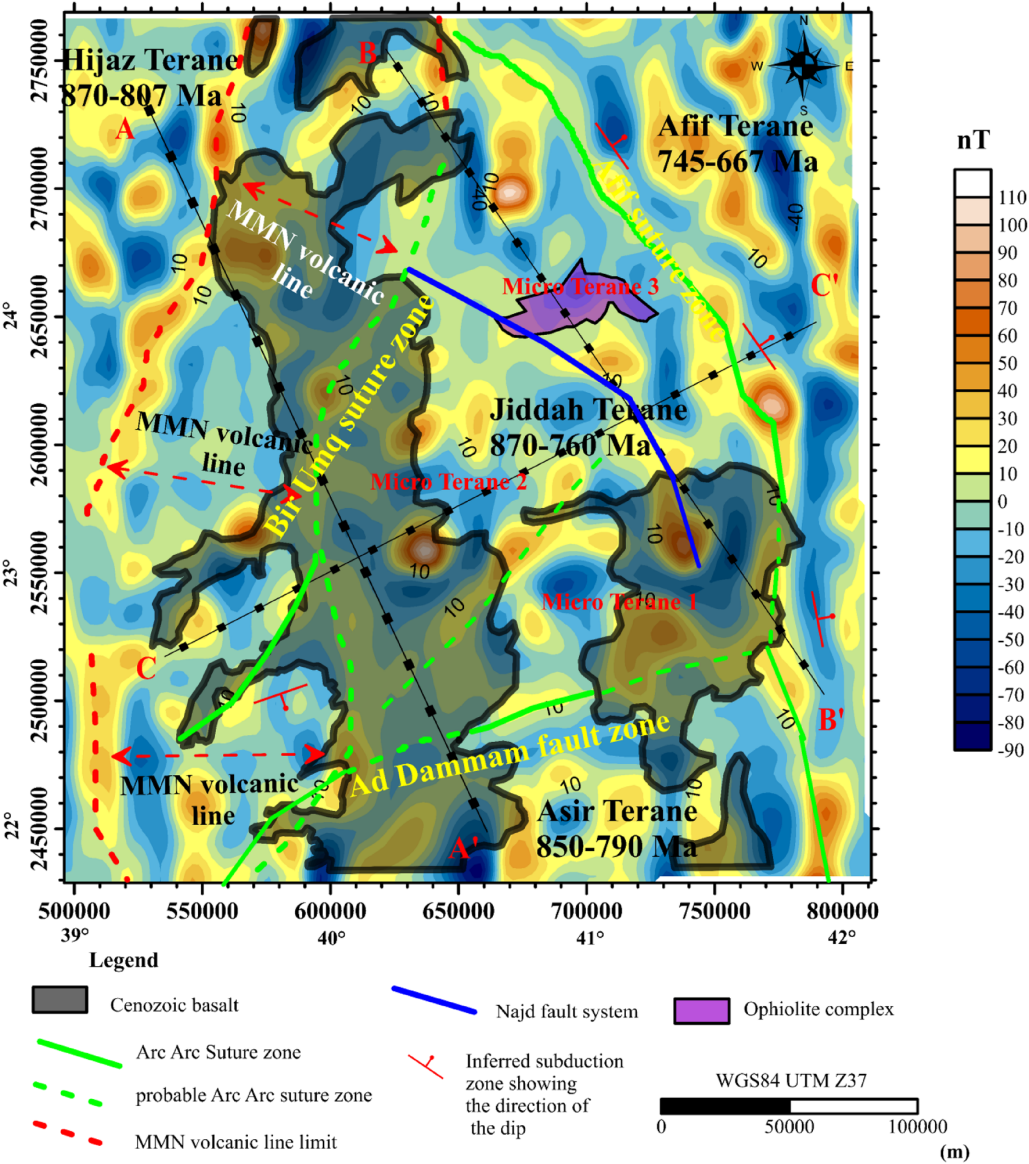
Interpreted aeromagnetic data of the Arabia shield, EMAG2 Magnetic anomaly Grid 2 arc minute resolution http://geomag.org/models/EMAG2, the transects A–A′, B–B′, C–C′ represents the thermal and seismic modeling profiles. Map generated using Golden Software Surfer V23 (https://support.goldensoftware.com/hc/en-us/articles/226806288-Download-my-software-online).

The gravity data utilized was extracted from the global static model, i.e., European Improved Gravity model of the Earth by New techniques (EIGEN 6C4)^41^, which is based on Gravity Recovery and Climatic Experiments (GRACE) observations^42^, Gravity field and steady-state Ocean Circulation Explorer (GOCE) satellites, and Earth Gravitational Model, EGM2008^43,44^. The EIGEN 6C4 is a comprehensive gravity field model and provides better gravity model resolution suitable for modeling the lithospheric blocks and structures than its previous counterparts. It can be extracted from the International Centre of Global Earth Model (ICGEM) portal. The resolution of the Bouguer anomaly grid that was generated based on spherical harmonics of the EIGEN 6C4 database was 0.15° (supple. Fig. 2). The isostatic gravity information utilized during the Moho depth estimations was acquired from the Bureau Gravimetric International. The resolution of the World Gravity Map, WGM 2012 model is 2 min. The crustal thickness utilized during the calculation of Airy compensation was 30 km, while 2.67 g/cm^3^ was used for density correction. The main objective of using the EIGEN 6C4 gravity data was to define the structural trends in the area based on the application of edge detection filters and to delineate the basement depth using the source parameter imaging technique. The WGM 2012 gravity data was used to map the Moho discontinuity by applying an empirical formula. Details are provided in the methodology section.

### Geothermal resource evaluation technique

The estimation of geothermal properties was performed by calculating the Curie depth, utilizing the spectral analysis of aeromagnetic data^8,45,46^. The centroid technique is one of the frequently employed methods of evaluating the crustal thickness and depth-to-bottom of the lithospheric mantle^45,47^. Like gravity and seismic refraction methods that can evaluate crustal layer boundaries where variations in the physical property i.e., density and elastic moduli exist respectively, magnetic susceptibility contrast can be used to distinguish magnetic responses of crust and lithospheric mantle layers. Reports of xenolith specimens of low geothermal regions within the lithospheric mantle showed the highest magnetic remanence^48,49^. These researchers also indicated that magnetic remanence can occur in areas where xenoliths sources showed temperatures < 600 °C. Hence, the centroid method in this study was applied to magnetic data to generate the Curie Point Depth (CPD). The technique was first adopted by^50,51^ and afterward^52^ and several other researchers also utilized this technique. Additionally, other researchers have adopted other techniques such as the de-fractal spectral analysis approach^53^ and the exponential technique^54^. The power density spectrum is defined as^55^

1 $$\phi_{\Delta T} \left( {k_{x} ,k_{y} } \right) = \phi_{M} \left( {k_{x} ,k_{y} } \right) \times F\left( {k_{x} ,k_{y} } \right)$$

2 $$F\left( {kx,ky} \right) = 4\pi^{2} C_{M}^{2} \left| {\theta_{M} } \right|^{2} \left| {\theta_{f} } \right|^{2} e^{{ - 2\left| k \right|Z_{t} }} \left( {1 - e^{{ - 2\left| k \right|\left( {Z_{b} - Z_{t} } \right)}} } \right)^{2}$$

where $\phi_{\Delta T}$ represents the total magnetic field power spectrum, *kx* and *ky* are respective wavenumber components in the *x* and* y* directions, *ϕ*_*M*_ is magnetization power density spectra, *C*_*M*_ is mathematical constant, $\theta_{M}$ and $\theta_{f}$ represents magnetic field and magnetization directions respectively, and *Z*_*b*_ and *Z*_*t*_ represents depths to bottom and top respectively of magnetized layers. Additionally, layer magnetization is usually represented as random functions of *x* and *y,* hence, the total field anomaly power density spectra *ϕ*_*M*_* (kx, ky)* is a constant function. More so, the azimuthally averaged power spectrum, $\phi \left( {\left| k \right|} \right)$ is given by

3 $$\phi \left( {\left| k \right|} \right) = Ae^{{ - 2\left| k \right|Z_{t} }} \left( {1 - e^{{ - 2\left| k \right|\left( {Z_{b} - Z_{t} } \right)}} } \right)^{2}$$

where *k* is the wavenumber and *A* is a constant. Defined the higher wavenumber segment of the power spectra curve slope, as top of the magnetic source (*Z*_*t*_) (Eq. 4)^56^,

4 $$ln\left( {\phi \left( {\left| k \right|} \right)^{\frac{1}{2}} } \right) = B_{1} - \left| k \right|Z_{t}$$

where *B*_*1*_ is a constant. Consequently, the low wavenumber part of the curve is used to calculate the depth to the centroid of the magnetic source (*Z*_*c*_) (Eq. 5).

5 $$ln\left( {\phi \left( {\left| k \right|} \right)^{\frac{1}{2}} } \right) = B_{2} - \left| k \right|Z_{c}$$

where *B*_*2*_ is a constant, while *Z*_*b*_ is defined with respect to *Z*_*t*_ and *Z*_*c*_ by^57^

6 $$Z_{b} = 2Z_{c} - Z_{t} .$$

Hence, the geothermal gradient (*dt/dz*) can approximately be determined using

7 $$\frac{dt}{{dz}} = \frac{{\theta_{c} }}{{Z_{b} }}$$

where *θ*_*c*_ represent the Curie temperature at atmospheric pressure, it is approximately 580 °C in igneous rocks^58–60^. The thermo-structure characteristics of the lithosphere can be defined by calculating the heat flow (Eq. 8), which depends on the geothermal gradient and variability of the thermal conductivity^61^. Therefore, the conductive heat flux (q) via a region of space is given by

8 $$q = \lambda \frac{dt}{{dz}} = \lambda \frac{{\theta_{c} }}{{Z_{b} }}$$

Due to lack in knowledge concerning the vertical and lateral variation of thermal conductivity^62^, λ, a mean value of 2.5 W/m/°C is taken to represent λ within the continental crust^62,63^. The error in the estimation of heat flow values due to the uncertainty in λ was observed to range between 5 and 15%. Crustal radioactive heat production is due to the disintegration of radionuclides, i.e., ^238^U, ^235^U, ^232^Th and ^40^K), and represents the contribution radiogenic elements in the continental crust heat flow production. Its empirical relationship is given by

9 $$A\left( z \right) = A_{0} e^{{\left( { - Z_{b} /D} \right)}} .$$

The value of radiogenic scaling depth (*D*) applied for the entire crust is 10 km^64^ while radiogenic heat coefficient at the surface^65^, *A*_0_ is taken as 1.5 mW/m^3^.

### Determination of the tectono-structural properties of the Western Arabian Shield

The use of derivatives of gravity and magnetic data for tectono-structural characterization has been a subject of debate within the scientific community. However, lots of research have proven its utility in solving problems related to terrane geodynamics^66–70^, potential field sources^71,72^, crustal imaging and mineral exploration^73–77^. More details on the historic evolution of these techniques can be found in^2^. Edge enhancement and detection filters that have been applied in many studies^78–85^ to interpret potential field data, were employed to define the structural styles within the vicinity under investigation. Such structures as distension and strike-slip zones, flower structures, faults and flexure zones have been mapped using these filters^9^. Developed and applied a novel technique based on improved logistic filter to better resolve the horizontal gradient derivatives^86^. Applied some edge enhancement filters to deduce the tectono-dynamic features of the Saskatoon region in Canada^87^. Use multi-approach to improve existing structural knowledge within Eastern Egypt, using analytic signal^88,89^, AS, total horizontal gradient, THG^90^, Theta map, TM^91^, tilt angle, TA^71^ and the logistic filter, LTHG^81^. Recently, an improved version of the logistic function filter was introduced to identify tectono-structural features in the Eastern Algerian Sahara^92^. Several investigations have been performed to improve the various techniques used to interpret tectono-dynamic systems worldwide^74,81,93–95^. The empirical relations of the edge enhancement filters applied in this study have been summarized in Table 1.

**Table 1:** The applied edge detection techniques to the EIGEN 6C4 data.

S /N	Name of technique	Authors	Mathematical formula
1	Analytic signal	89	$\left| {AS} \right| = \sqrt {\left( {\frac{\partial f}{{\partial x}}} \right)^{2} + \left( {\frac{\partial f}{{\partial y}}} \right)^{2} + \left( {\frac{\partial f}{{\partial z}}} \right)^{2} }$
2	Tilt Angle	71	$TA = atan\left[ {\frac{{\left( {\frac{\partial f}{{\partial z}}} \right)}}{{\sqrt {\left( {\frac{\partial f}{{\partial x}}} \right)^{2} + \left( {\frac{\partial f}{{\partial y}}} \right)^{2} } }}} \right]$
3	Theta map	_91_	$THETA = a\,cos\,cos \left( {\frac{THG}{{AS}}} \right)$
4	The total horizontal derivatives of the tilt angle	90	$THG = \sqrt {\left( {\frac{\partial f}{{\partial x}}} \right)^{2} + \left( {\frac{\partial f}{{\partial y}}} \right)^{2} }$
_5_	Tilt angle of horizontal gradient	49	$TAHG = a\,tan\,tan \left[ {\frac{{\left( {\frac{\partial THG}{{\partial z}}} \right)}}{{\sqrt {\left( {\frac{\partial THG}{{\partial x}}} \right)^{2} + \left( {\frac{\partial THG}{{\partial y}}} \right)^{2} } }}} \right]$
_6_	Logisticfunction of total horizontal gradient	79	$LTHG = \left[ {1 + exp\left( { - \frac{{\frac{\partial THG}{{\partial z}}}}{{\sqrt {\left( {\frac{\partial THG}{{\partial x}}} \right)^{2} + \left( {\frac{\partial THG}{{\partial y}}} \right)^{2} } }}} \right)} \right]^{ - \alpha }$
7	Modified theta map	74	$MTM = cos^{ - 1} \left( {\frac{{\sqrt {f_{zx}^{2} + f_{zy}^{2} } }}{{\sqrt {f_{zx}^{2} + f_{zy}^{2} + \left( {\frac{{f_{z} }}{h*p}} \right)^{2} } }}} \right)$
8	Improved logistic function of total horizontal gradient	92	$LTHG = \left[ {1 + exp\left( { - \frac{{\frac{\partial ITHG}{{\partial z}}}}{{\sqrt {\left( {\frac{\partial ITHG}{{\partial x}}} \right)^{2} + y\left( {\frac{\partial ITHG}{{\partial y}}} \right)^{2} } }}} \right)} \right]^{ - \alpha }$
9	Normalized tilt angle	93	$NDX = a\,tan\,tan \left[ {\frac{THG}{{\left| {\frac{\partial f}{{\partial z}}} \right|}}} \right]$

### Crustal thickness estimation

Gravity anomalies provide useful information for estimating crustal density and thickness^96–99^. Several researchers have developed empirical equations that are available in relevant literatures, to define crustal thickness^93,100,101^. Empirical relationship that relates the Moho depth $\left({H}_{m}\right)$ expressed in kilometer and regional isostatic gravity anomaly $\left(f\right)$ expressed in milligal was utilized (Eq. 10)^102^.

10 $$H_{m} = 32 - \left( {0.08 \times f} \right)$$

By this study, we seek to characterize and infer the origin of geothermal flow and its relation with the structural dynamics, by examining the magnetic and gravity information of the WAS. The basement depth was calculated using the source parameter imaging algorithm (SPI) based on the calculation of local wavenumber of analytic signal. Detailed theoretical aspects of the method can be found in^103,104^.

### Estimation of the Lithosphere-Asthenosphere Boundary (LAB)

The depth to the LAB (*Z*_*L*_) is calculated using empirical relation^105^, where* E* is the topography and *N* the geoid, the LAB is expressed as

11 $$Z_{L} = \frac{1}{{\rho_{a} - \rho_{c} }}\left( {\kappa + \sqrt {\frac{{\rho_{m} - \rho_{c} }}{{\rho_{m} - \rho_{a} }}(\kappa^{2} - \left( {\rho_{a} - \rho_{c} } \right)\left[ {E^{2} \left( {\rho_{w} - \rho_{c} } \right) + Zmax^{2} \rho_{a} + \left( {N - N0} \right)\frac{g}{\pi G}} \right]} } \right)$$

The initial constants used to represent the crustal and mantle densities were $\rho_{c}$ = *2.7* and $\rho_{m}$ = *3.2* g/cm^3^ respectively. The asthenospheric density, $\rho_{a}$ = *3.24* g/cm^3^, water density, $\rho_{w}$ = *1* g/cm^3^, Z_max_ is the isostatic compensation level taken as 300 km, g is the terrestrial gravitational acceleration and G is the gravitational constant^105^.

12 $$\kappa = \rho_{a} L_{0} + E\left( {\rho_{c} - \rho_{w} } \right)$$

where *L*_*0*_ is 2320 m and represents the free asthenospheric depth^105^*.*

13 $$N_{0c} \left( {E,N} \right) = \frac{\pi G}{g}\left( {E\left( {\rho_{w} - \rho_{c} } \right) + Z^{2} \frac{{\kappa^{2} }}{{\rho_{a} - \rho_{c} }}} \right) + N$$

where *N*_*0*_ is considered as a reference level required to adjust the *0* level of the geoidal anomalies.

### 2D thermal and seismic modeling

The implementation of 2D numerical modeling of temperature, densities, P- and S-wave velocities utilized here are documented^106^. Generally, the modeling is based on petrophysical and mineral physics and provides a means of estimating relevant thermophysical proprieties of the lithospheric mantle^107,108^. Thermal conductivity is assumed to be constant within the crust while it depends on pressure–temperature variations within the mantle^109^. Gibbs free-energy minimization algorithm is used to calculate the stable mineral assemblage and phase equilibrium in the lithospheric mantle. Densities were computed by solving the iterative fourth order Birch-Murnaghan equations. The bulk rock densities were results of the arithmetic mean of the phases weighted by the volumetric fraction^108^. The elastic moduli were used to calculate P- and S-wave velocity distribution from each end-member mineral and the bulk rock density at desired temperature and pressure^107^. Additionally, to achieve the temperature, density and P- and S-wave velocity models, three representative transects were used. The crustal-mantle structure was constructed using the results in the previous sections (i.e., heat flow, basement depth, Moho, LAB layers) as inputs in LitMod 2D.

The profiles A–A′ and B–B′ cut across Hijaz, Jiddah and Asir terranes. More so, the third transect is constrained by the Arabian craton to the East and the Red Sea rift to the West (Fig. 2). These transects were constrained by results from previous seismic and thermal studies within the ANS^110–112^. The observable geophysical data used for modeling were; free air gravity, Bouguer gravity, topographic and geoidal information. The seismic-thermal-density architecture of the lithospheric-mantle and asthenosphere was achieved by simultaneously fitting long wavelength components of geophysical data along the previously chosen transects.

## Results

### Magnetic data interpretation

To overcome the dipolar effects due to the unknown factors that can influence the magnetic field data, reduction-to-the-magnetic pole (RTP) was performed^113^ and thereafter, other preprocessing such as upward continuation and anomaly separation were performed. For the purpose of this thematic, we correlated magnetics with the geologic trends provided from previous studies. Magnetic anomaly values range between − 90 and 110 nT, with alternate positive and negative anomalies resulting from compressional and extensional events of the amalgamated terranes. Hijaz and Jiddah terranes are separated by the volcanic line, i.e., Makkah-Madinah-Nafud (MMN), represented in the magnetic map by two positive magnetic borders (> 30 nT). High magnetic anomalies with predominant NE–SW and NW–SE orientations cover the majority of terranes within the investigated area. Magnetic highs resulting from the ascension of late to post tectonic granitoids, were observed within the Hijaz terrane. The Jiddah terrane represents an important variety of tectonic structures that have close proximity to magnetic anomalous zones. Highest magnetic values represent suture zones and contact between the amalgamated terranes, while unmapped suture zones were observed under the basaltic lava and separates the basalt domain into micro-terranes. The Harrats (i.e., Cenozoic lava) and the Furayh basin presented no specific magnetic anomaly signature pattern, i.e., both positive and negative magnetic anomalies that range between − 70 and 90 nT were observed within the basin. The Afif suture that separates the Afif and Jiddah terranes is characterized by magnetic high that range between 30 and 70 nT. Towards the southern segment of the study area, an extension of the Cenozoic basalt covered probable suture zones, characterized by magnetic highs and represented in Fig. 2 by green broken lines. These sutures are the results of the collision between the Asir and Jiddah terranes. The magnetic lows correspond to tectonic features such graben or basement deformations that are close to the suture limits. Also, these low values represent subduction tectonic zones^114^. The segment of the residual magnetic map where this feature is located has been marked by red oriented arrows.

### Evaluation of thermal properties

Studies have shown that the window size controls the efficacy of the spectral analysis^57,77,115^. Several researchers have estimated the Z_b_ using different variants. Grid dimensions of five times the Curie depth have been applied^55^. Window size of 77 × 77 km have been used^115^. Also, a grid of 200 × 200 km was used to determine the Curie depth in east and south Asia^57^. Suggested 60 × 60 km while studying the Red Sea coast^116^. Window size of 75 × 75 km was used to study the Eastern Algerian Sahara craton^77^.

**Figure 3:**
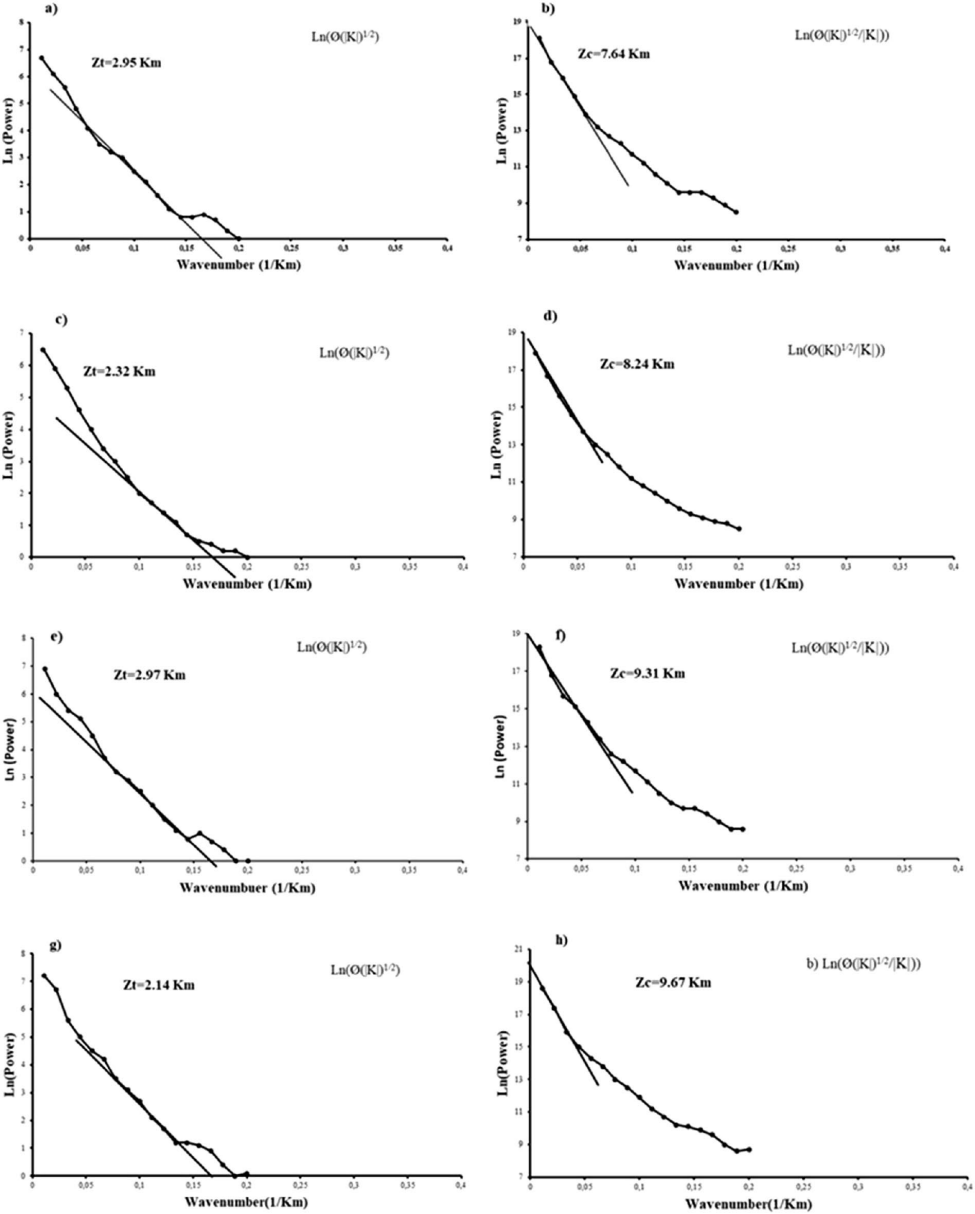
Examples of spectral analysis depth estimation: (**a**, **b**) Block 6; (**c**, **d**) Block 11; (**e**, **f**) Block 37; (**g**, **h**) Block 55.

An approximate value of 100 × 100 km was used to estimate the Curie depth within the central Red Sea^62^. A window size of 1000 × 1000 km was employed to evaluate the geothermal potentials of the African continent^8^. The window size employed in this paper is 75 × 75 km in dimension and was used to define Z_b_ due to similarities in the crustal architecture and the geologic events, to that of the Algerian Saharan platform. Additionally, based on geological evidences of the tectonic fields within the WAS, the optimum window size was carefully arrived at, after several trials with larger window sizes. This was done to permit certain components of the spectrum with a view of improving the spatial resolution of the method. Applying the USGS module in Geosoft Oasis Montaj software, 80 blocks were calculated. Details of the technique and data treatments are documented^77^. The spectra analysis (Fig. 3) technique was used to calculate the center and top of the magnetic anomaly at each window. The Curie depth ranges between − 9.76 and − 25.7 km and the lower values were observed in proximity to the Red Sea coast and suture zone (supple. Fig. 3). The windows covering the Arabian flank reveal Curie depth values that were approximately the same (i.e., using the centroid and diffractal spectral analysis). This indicates that the selection of the spectra slope is related to the deepest magnetic layer over the multi-magnetic layers encountered especially within the continental crust. However, estimation of depths to magnetic sources using power spectral technique is influenced by the resolution of the magnetic data. Detailed discussion on the effect of variation in resolution of onshore and offshore magnetic data and explained how it can influence the estimation of Curie depth along the Red Sea rift and its Eastern and Western flanks have been provided by^62^. Previous estimates of the Curie depth along the Arabian flank of the rift gave values of that range between 16 and 18 km and ~ 8 km at the center of the Red Sea, using the centroid method^62^. By approximation, Curie depth values > 17 km were assumed to be overestimated based on our conclusions. Also, the data used for this study is high resolution as it is an integration of several grids of magnetic data. In order to validate the Curie depth estimates of our work, we compared our results with values observed^62^ and the error estimate range between 10 and 15%. However, the Curie depth estimation error due to the application of the centroid method is dependent on the geology of the area, the expertise of the interpreter and the resolution of the magnetic data used.

**Figure 4:**
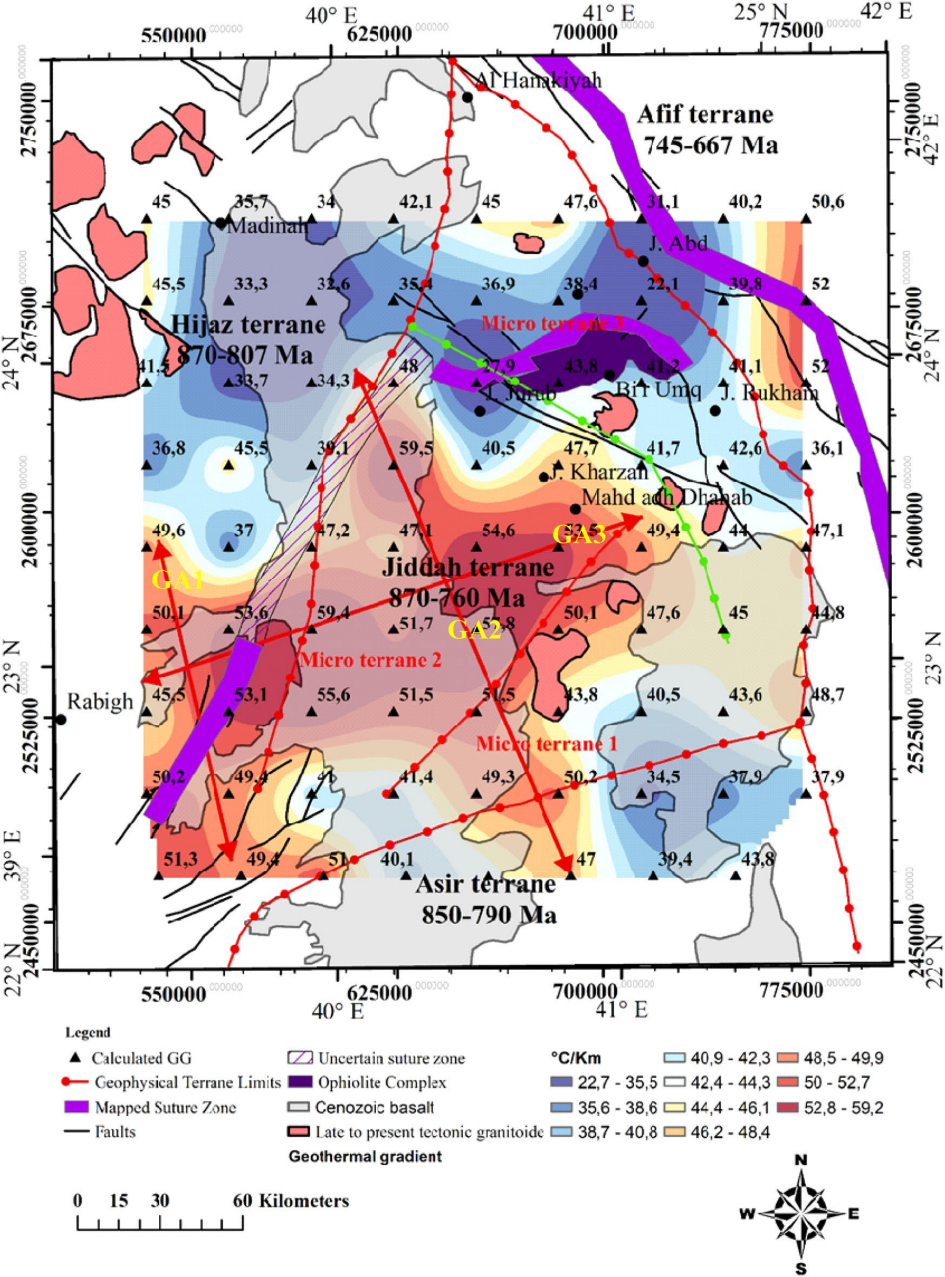
Spatial distribution of geothermal gradient derived from aeromagnetic data applying Centroid method. Map generated using ArcGIS Pro (https://pro.arcgis.com/en/pro-app/latest/get-started/download-arcgis-pro.htm).

**Figure 5:**
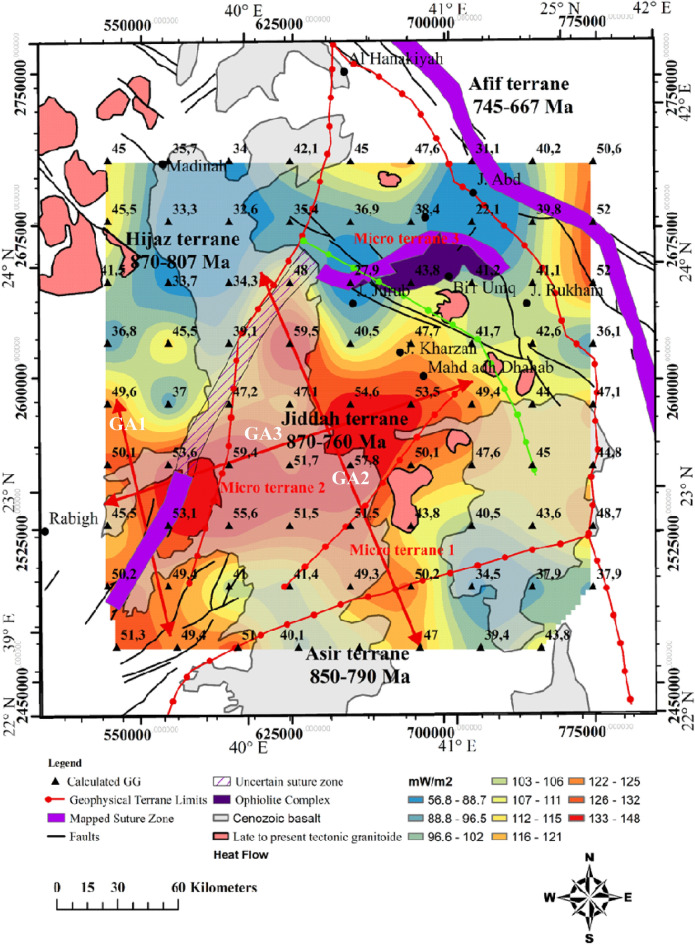
Spatial distribution of heat flow derived from the application of the centroid method on aeromagnetic data of the area. Map generated using ArcGIS Pro (https://pro.arcgis.com/en/pro-app/latest/get-started/download-arcgis-pro.htm).

The Curie temperature was assumed to be 580 °C^58–60^, and this value offers a means of calculating geothermal gradient across the entire area. Portions of the study where geothermal gradients were conspicuous, were identified (i.e., along the NW–SE and E–W directions), crossing the Cenozoic basalt and arc rocks of the Jiddah terrane (Fig. 4). These results indicate that usioing the magnetic data can provide information on the thermo-structural trends within the middle and lower crust. Correlation of these findings with previous studies^21^ elucidate significant contrast in geothermal gradient values, with the highest gradients occurring within the GA1, GA2 and GA3 (Fig. 4) thermal axis (~ 46 °C to 49 °C/km). The lowest gradients were noticeable around the northern parts (i.e., trends in the E–W direction) crossing the Furayh sedimentary basin (22.7 °C to 38 °C/km). Relatively high heat flow average (i.e., 110 mW/m^2^) was deduced from the EMAG2 data by applying the centroid method within the area. This average, when compared with observations from the Northern Algerian Sahara (i.e. 89 mW/m^2^)^77^ reveals significant differences probably due to the nature of tectonic structure, which was considered to be stable within the Northern Algerian Sahara. However, both observations exceeded the international mean value^117^ of 87 mW/m^2^. The spatial heat flow map reveals considerable regional variations related to short and long wavelengths anomalies, attributable to the structural styles of the crust (Fig. 5). There were E–W and NW–SE zonations that relates directly to the actual structures from deep lithospheric rifting of the Red Sea. The NW–SE (GA1 and GA3) thermal axis (Fig. 5) yields high heat flow (115–131 mW/m^2^). The GA3 E–W axis (Fig. 5), typically displayed highest heat flow values (~ 120 to 148 mW/m^2^). Generally, these heat flow values agreed with measured values (i.e., 100–200 mW/m^2^) reported for the area^62^. Low to moderate heat flow values were observed in Furayh sedimentary basin that range between 56 and 100 mW/m^2^. These values were justified by coupled lithospheric processes and regional asthenospheric flows such as crustal thinning and decompression melting, and locally, by a myriad of late tectonic events within the Bi’rUmq suture.

### Evaluation of radiogenic heat

**Figure 6:**
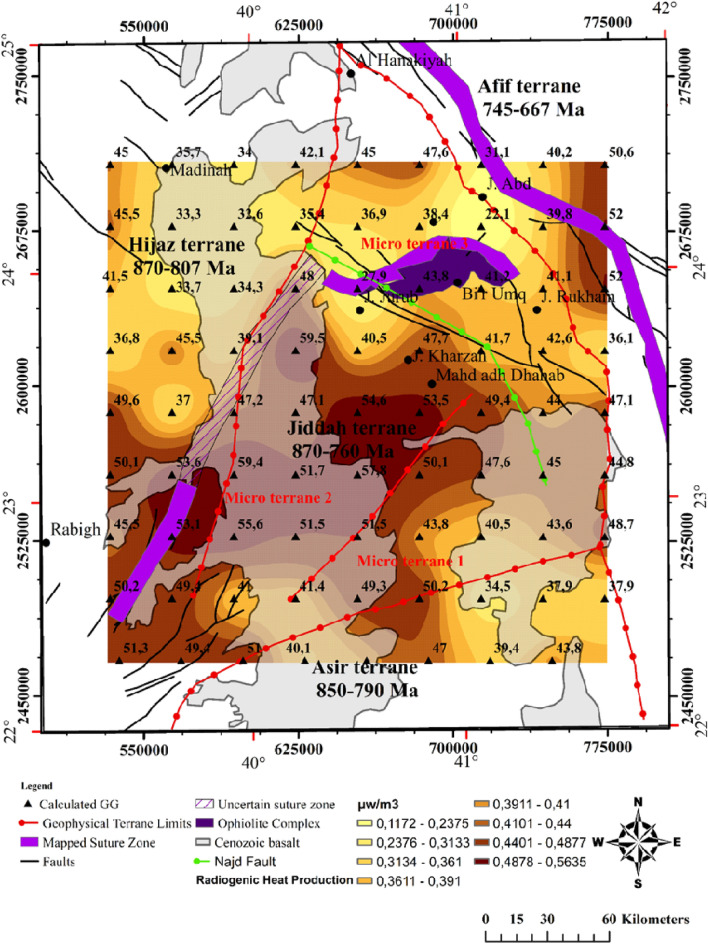
Estimated radiogenic heat production for depth of 10 km from interpretation of magnetic basement depth estimated using source parameter imaging of the gravity data. Map generated using ArcGIS Pro (https://pro.arcgis.com/en/pro-app/latest/get-started/download-arcgis-pro.htm).

The radiogenic heat production in southwestern Saudi Arabia as estimated using Eq. (10), range between 0.12 and 0.56 μW/m^3^ (Fig. 6). Relatively high values were observed around the center, i.e., the Jiddah terrane, while lowest values were noticed in areas where the Curie Point Depth (CPD) occurs at lower depths^77^. Figure 6 shows the heat potential produced by radiogenic elements in radiogenic depth scaling^118^. Generally, heat production is controlled by the age and type of rocks as well as regional tectonic history^119^. Heat production in granites from different locations in the world were observed to range between 0.2 and 8.1 μW/m^3^. Different values of radiogenic heat, i.e. 1.82–4.37 μW/m^3^ and 0.32 to 8.04 μW/m^3^ from granitic rocks in the northeastern and southeastern Arabian shield respectively, have been reported^120^. The differences in the range of values can be attributed to the melting process at the lower crust which affects the distribution of radioelements. The Cenozoic intrusions are more depleted in radioelements than their counterparts of mid-Proterozoic times^121^. Table 2 shows a summary of results of top, center and bottom depth of magnetic anomaly, heat flow, radiogenic heat production and geothermal gradient for some blocks within the study area.

**Table 2:** Examples of calculated Curie depth, geothermal gradient, heat flow and radiogenic heat production applying the centroid method.

Centroid no	X (m)	Y (m)	Z_t_ (km)	Z_c_ (km)	Z_B_ (km)	Geothermal gradient (°C/km)	Heat flow (mW/m^2^)	Radiogenic heat production (μW/m^3^)
1	538,024.58	2,467,394.39	− 2.31	− 6.81	− 11.31	51.30	128.25	0.48
5	658,024.58	2,467,394.39	− 2.70	− 8.06	− 13.41	43.23	108.08	0.39
7	718,024.58	2,467,394.39	− 1.53	− 6.88	− 12.22	39.36	98.40	0.34
10	563,587.16	2,497,394.39	− 2.59	− 7.16	− 11.73	49.43	123.58	0.46
13	653,587.16	2,497,394.39	− 2.56	− 7.16	− 11.77	49.27	123.17	0.46
15	713,587.16	2,497,394.39	− 3.26	− 10.03	− 16.80	34.52	86.31	0.28
20	593,587.16	2,527,394.39	− 2.47	− 6.45	− 10.43	55.61	139.02	0.53
24	713,587.16	2,527,394.39	− 2.87	− 8.60	− 14.33	40.47	101.18	0.36
30	623,587.16	2,557,394.39	− 2.76	− 6.99	− 11.21	51.74	129.36	0.49
35	773,587.16	2,557,394.39	− 2.46	− 7.70	− 12.95	44.79	111.97	0.42
40	653,587.16	2,587,394.39	− 2.63	− 6.63	− 10.63	54.56	136.41	0.52
45	533,587.16	2,617,394.39	− 1.43	− 8.60	− 15.76	36.79	91.98	0.31
50	683,587.16	2,617,394.39	− 3.13	− 7.64	− 12.15	47.73	119.32	0.44
55	563,587.16	2,647,394.39	− 2.15	− 9.67	− 17.19	33.72	84.31	0.27
60	713,587.16	2,647,394.39	− 3.14	− 8.60	− 14.06	41.24	103.11	0.37
65	593,587.16	2,677,394.39	− 2.29	− 10.03	− 17.77	32.63	81.59	0.25
70	743,587.16	2,677,394.39	− 3.10	− 8.84	− 14.57	39.80	99.52	0.35
75	623,587.16	2,707,394.39	− 2.48	− 8.12	− 13.76	42.12	105.32	0.38
80	773,587.16	2,707,394.39	− 3.22	− 7.34	− 11.64	50.59	126.74	0.48

### Delineation of tectono-structural trends

**Figure 7:**
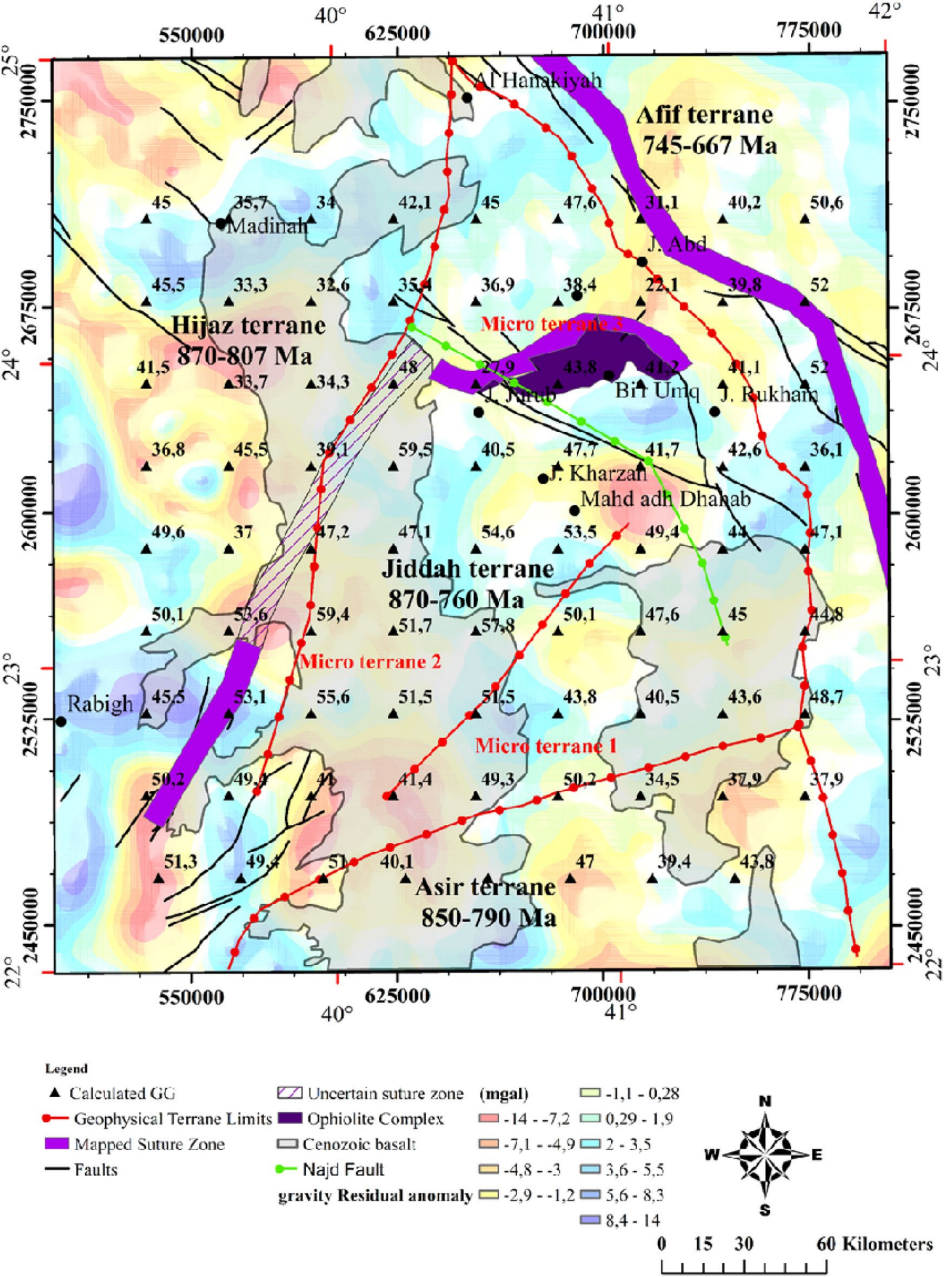
The Gravimetric map of the Western Arabian Shield, from EIGEN 6C4 database, (http://icgem.gfz-potsdam.de/tom_longtime) updated Terrane limits from interpretation of magnetic interpretation marked by Red bold lines. Map generated using ArcGIS Pro (https://pro.arcgis.com/en/pro-app/latest/get-started/download-arcgis-pro.htm).

**Figure 8:**
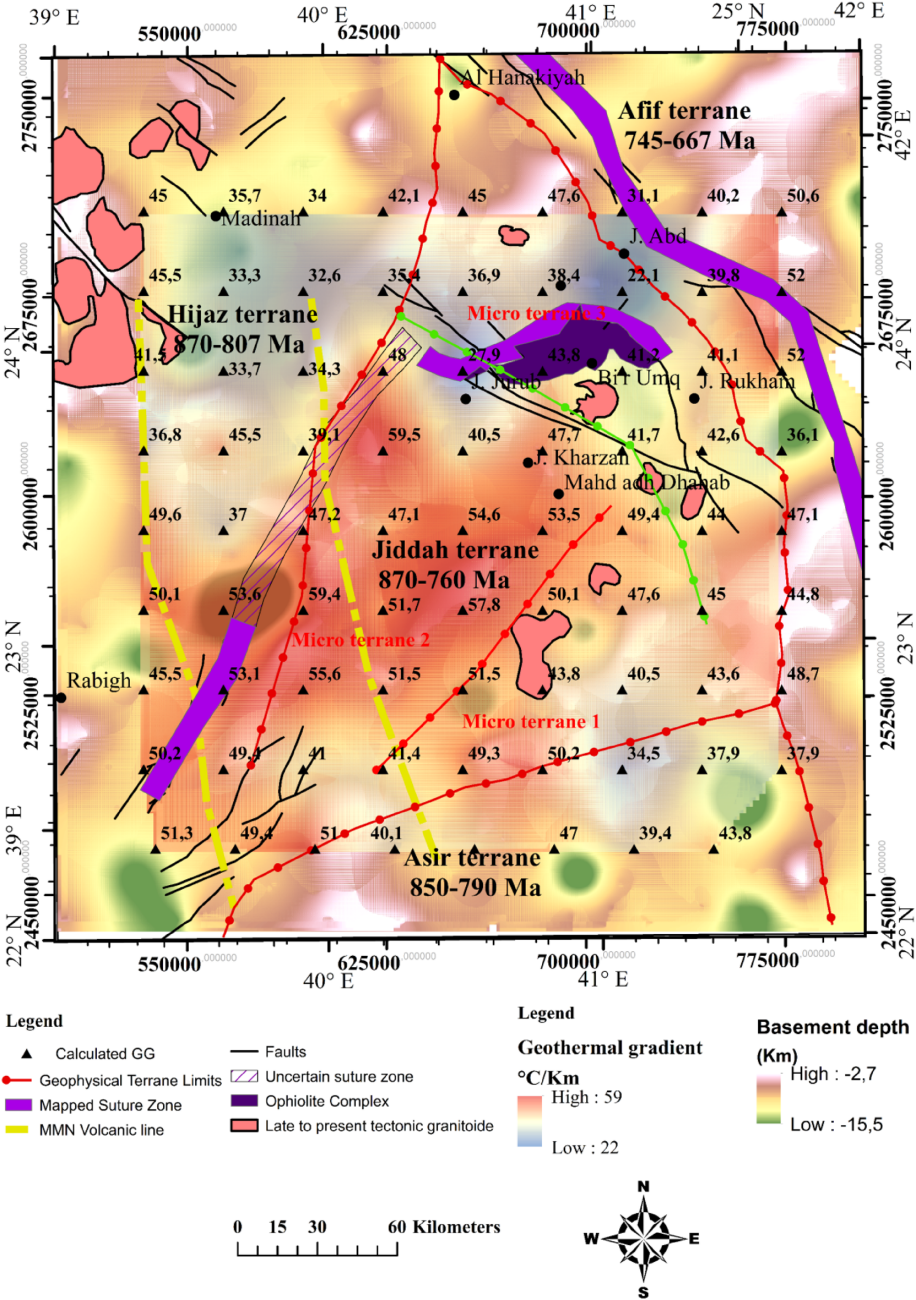
Upper crust structure, obtained from gravimetric data inversion, superimposed on the geothermal gradient distribution, the red arrows show geothermal axis and the potential zones. Map generated using ArcGIS Pro (https://pro.arcgis.com/en/pro-app/latest/get-started/download-arcgis-pro.htm).

**Figure 9:**
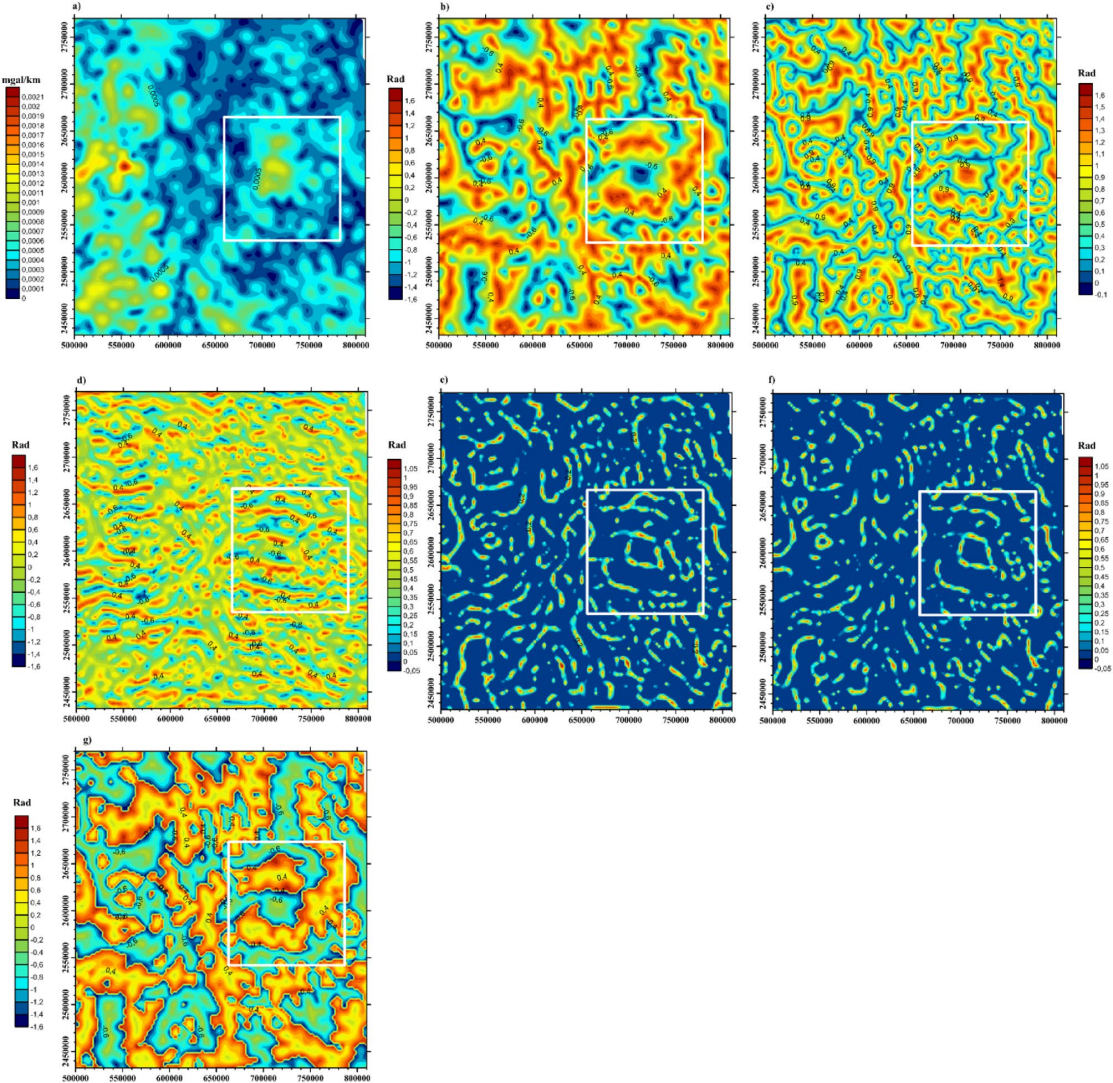
Comparative results of edge detection techniques for structural mapping, white rectangle represents the limits of negative flower structure: (**a**) AS; (**b**) TA; (**c**) TM; (**d**) TAHG; (**e**) LTHG; (**f**) ILTHG; and (**g**) TDX. Map generated using Golden Software Surfer V23 (https://support.goldensoftware.com/hc/en-us/articles/226806288-Download-my-software-online).

**Figure 10:**
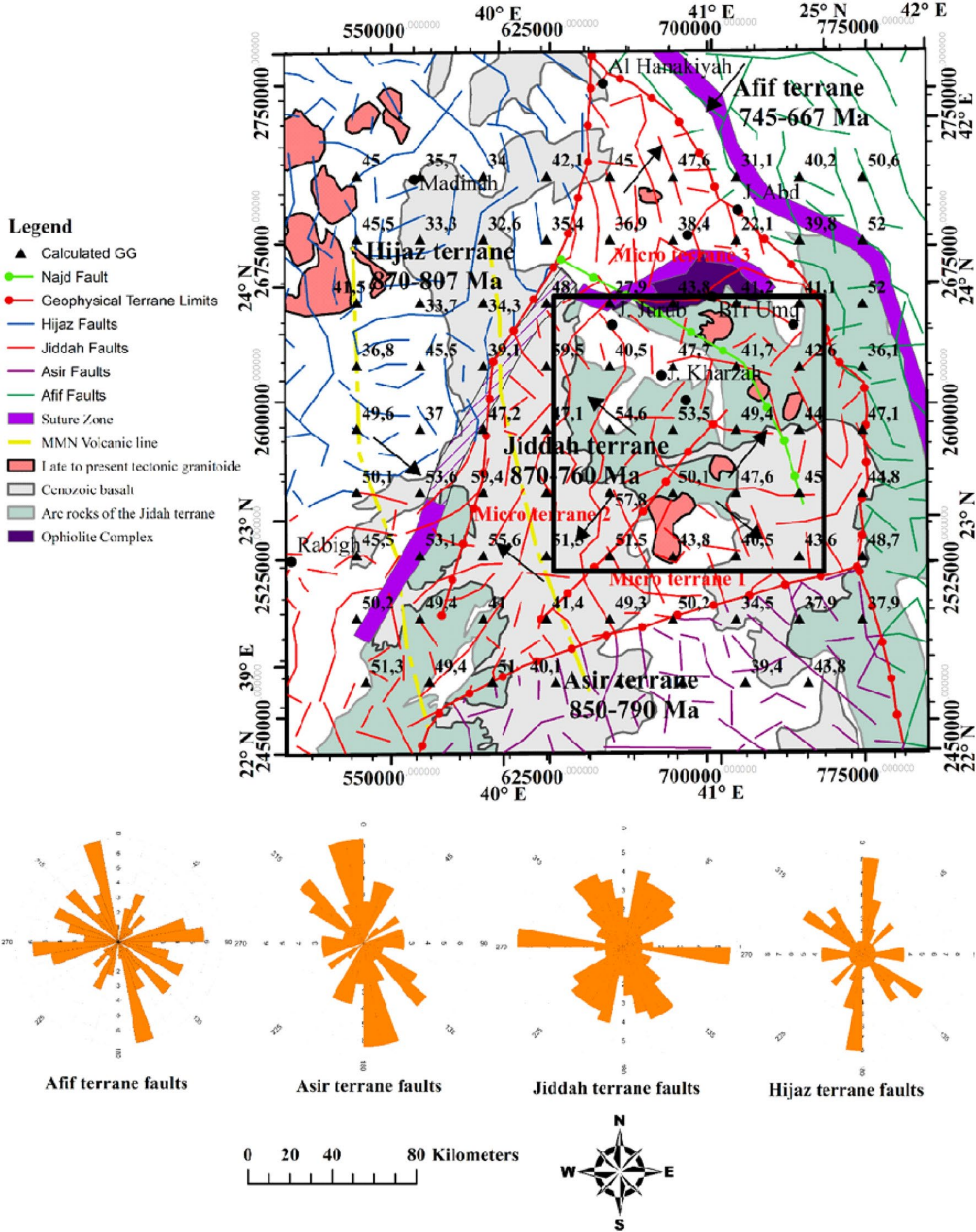
Synthetic structural map of the study area obtained using combined edge detection techniques, showing the major tectono-structural units of western Saudi Arabia. Red broken lines represent suture zone and terrane limits with strike-slip zone, black rectangle represent the limits of negative flower structure. Map generated using ArcGIS Pro (https://pro.arcgis.com/en/pro-app/latest/get-started/download-arcgis-pro.htm).

The Bouguer data is filtered from the regional component (supple. Fig. 2), and Fig. 7 shows the distribution of the residual potential field. Generally, the anomalies range between − 14 to + 14 mGal. Several of these structural orientations were not observed in the analytic signal map but were conspicuously observed as continuous false edges in the tilt angle, theta map, and tilt derivative x-direction maps (Figs. 8 and 9). Analysis of the structural styles (Fig. 10) revealed that the area is predominantly composed of several tectonic structural trends. However, the NE–SW, NW–SE, NNE–SSW and E–W structural trends were rare. The NW–SE faults affected the upper crust formation as normal vertical faults or sinistral strike-slip faults indicating compression and/or distortion processes^122^. The comparison of edge enhancement techniques enabled the identification of certain distinct structural styles that indicates strike-slip movements. In the study of tectonics, flower structures are distinctive characteristics of wrench zones. These structures typify zones of strike-slip faults^123^. Near the Afif suture zone, negative flower structure was observed around the Jiddah terrane (Fig. 9). The identified flower structures were compared with conventional models^124^, and it revealed shallow synform with strands that spread upward resulting from normal fault structures, having outward convex configuration, that converges with a central subvertical core at depth. Similar flower structures have been observed in Bohai Bay basin, China and Ougarta, southwestern Algerian^9,123^. The structure map of the WAS like its Ougarta counterpart, show evidence of transtensional divergent movements^9^.

**Figure 11:**
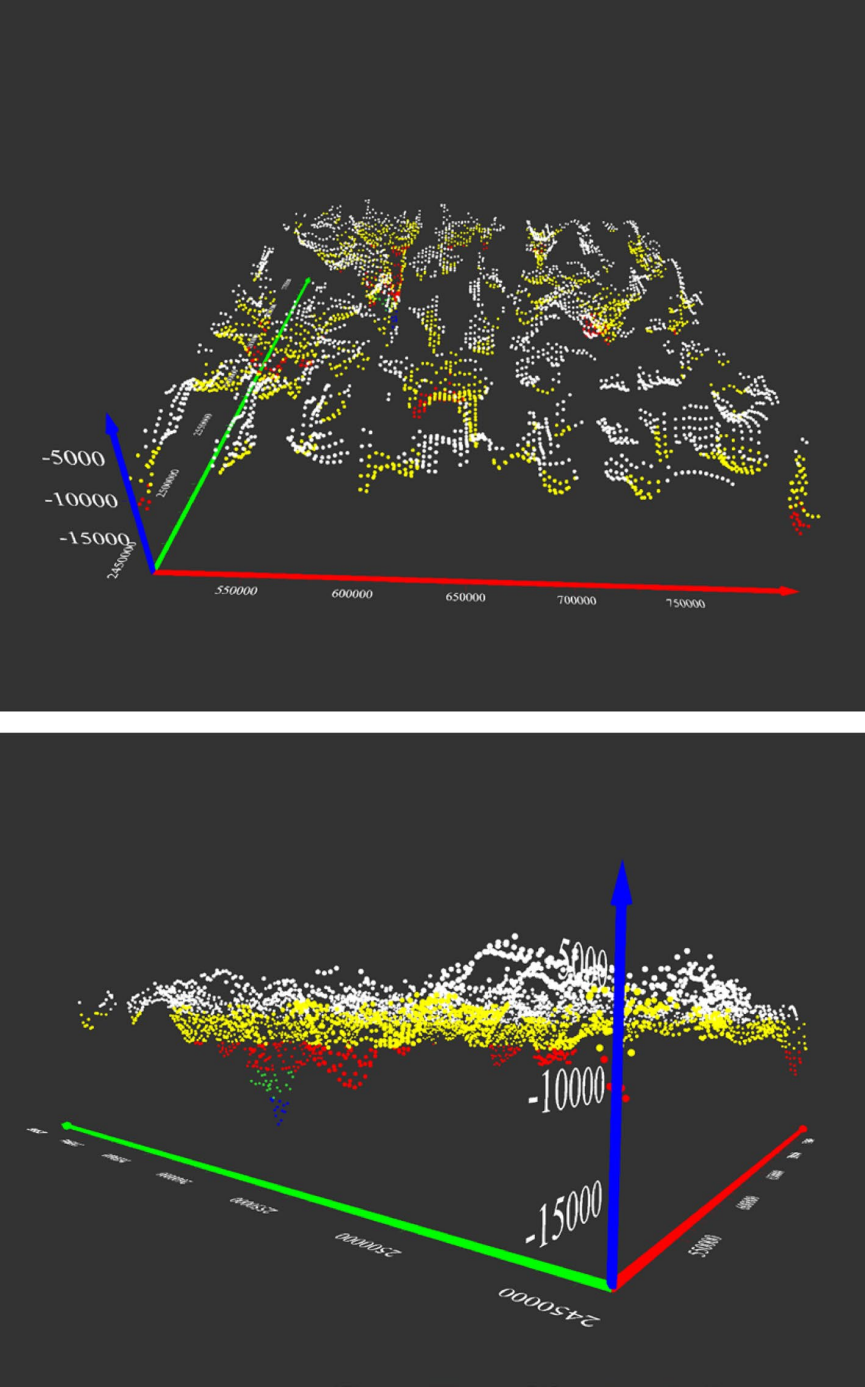
Euler depth solution derived from 3D Euler deconvolution, using Euler equation and structural index SI = 0. Map generated using Golden Software Surfer V23 (https://support.goldensoftware.com/hc/en-us/articles/226806288-Download-my-software-online).

Figure 11 shows an overlay of the 3D Euler deconvolution solutions on the calculated geothermal gradient map. The structural index for dyke/contact for the gravity data was zero. Deep seated fault structures that NE-SW and NW–SE trends were mapped at depths further than 6.5 km and are similar to those reported^125^ along Oko shear zone. The dominant faults represent the structural continuity of the Najd fault that influences sedimentary thickness in the area. This major fault system is characterized by several secondary linear fault extensions along its border within the sedimentary basins^21^. Previous geophysical studies mentioned that the Najd fault system (640–560 Ma) is an expansive zone of NW trending brittle-ductile shears that extends along 1100 km by length and 300 km by width. It is often considered as one of the prominent late Proterozoic structures in the ANS^122^. This system is thought to result from the collision of the westerly ANS and the easterly Arayn microplate^125^.

### Moho depth estimation

**Figure 12:**
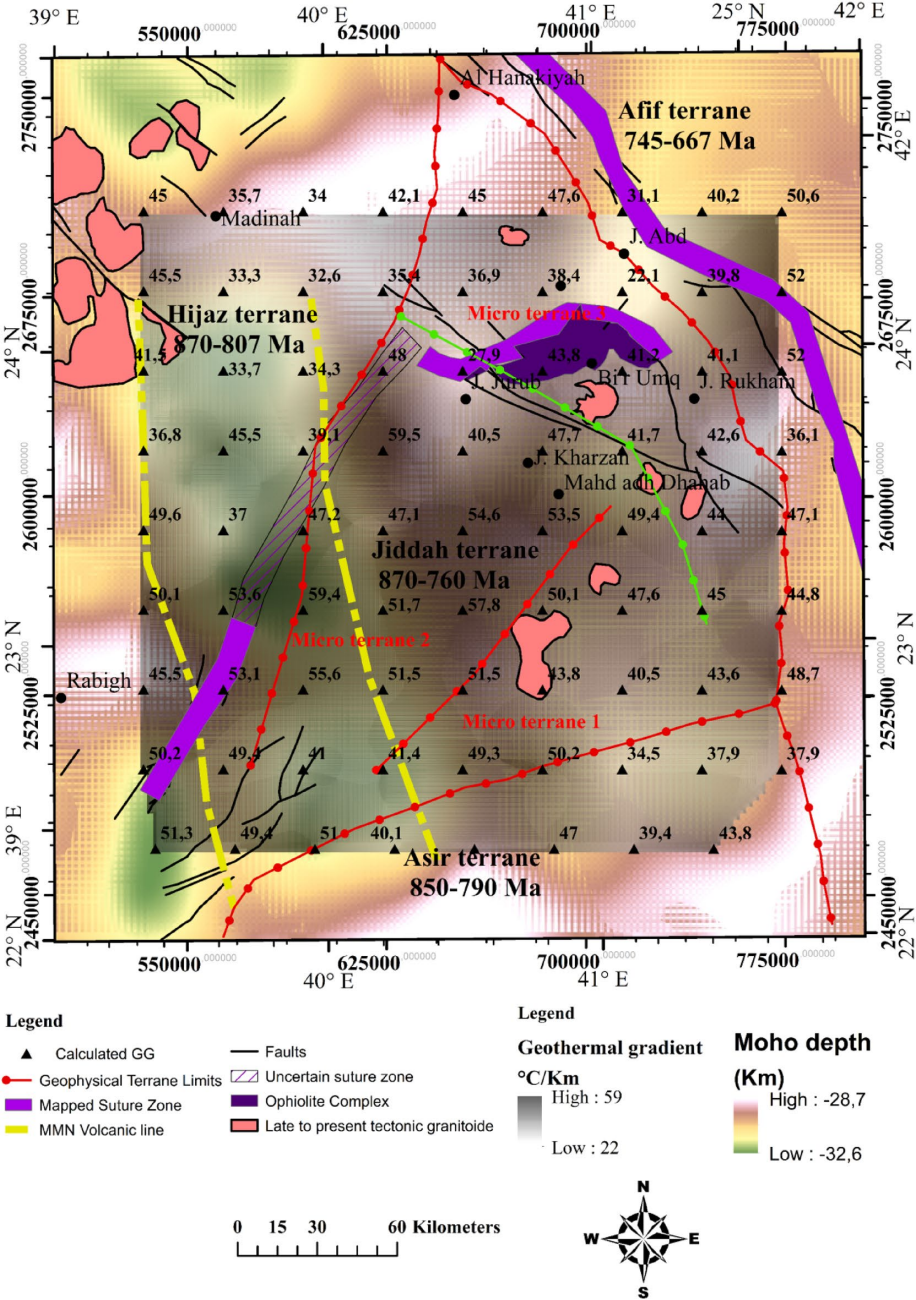
Lower crust architecture derived from EIGEN 6C4 gravity data, correlated with estimated geothermal gradients and regional fault structures derived from 3D Euler Deconvolution (> 6.5 km depth). Map generated using ArcGIS Pro (https://pro.arcgis.com/en/pro-app/latest/get-started/download-arcgis-pro.htm).

Several studies mentioned different depths of the Moho discontinuity within the Arabian province. Studies reported 38 km and 22–25 km values respectively, for the Moho depth in WAS, while the central and southern limits of the shield were reported as 40–45 km and 35 km respectively^126,127^. Average depth beneath the Arabian province has been reported to be approximately 39 km and 35–38 km respectively^128,129^. Seismic receiver function estimated the crustal thickness to vary from 25 to 45 km^130^. The Hijaz terrane is characterized by value of 32.5 km, while the Harrat Rahat show values that range between 32.5 and 37.5, Jeddah terrane (27.5–40 km), Asir terrane (27.5–45 km), and Afif terrane (35–37.5 km). Recently^131^ estimated crustal thickness within the study area to be between 17 and 35 km. In this study, we approximate the maximum crustal thickness to be 32.6 km at the western limit of Furayh basin and the Cenozoic basalt of Harrat Rahat representing probably a subduction area or strike-slip deep fault covered by thick deposits or the extension of the MMN volcanic line (Fig. 12). The crust attained minimum thickness at the eastern part of Furayh Basin and Harrat Hirmah, i.e., where the Moho reaches 28 km. Similar values were calculated within the southeastern flank. Beneath Jiddah terrane and Ghamir basin, the Moho is between 31 and 30 km in depth.

### Estimation of the LAB

**Figure 13:**
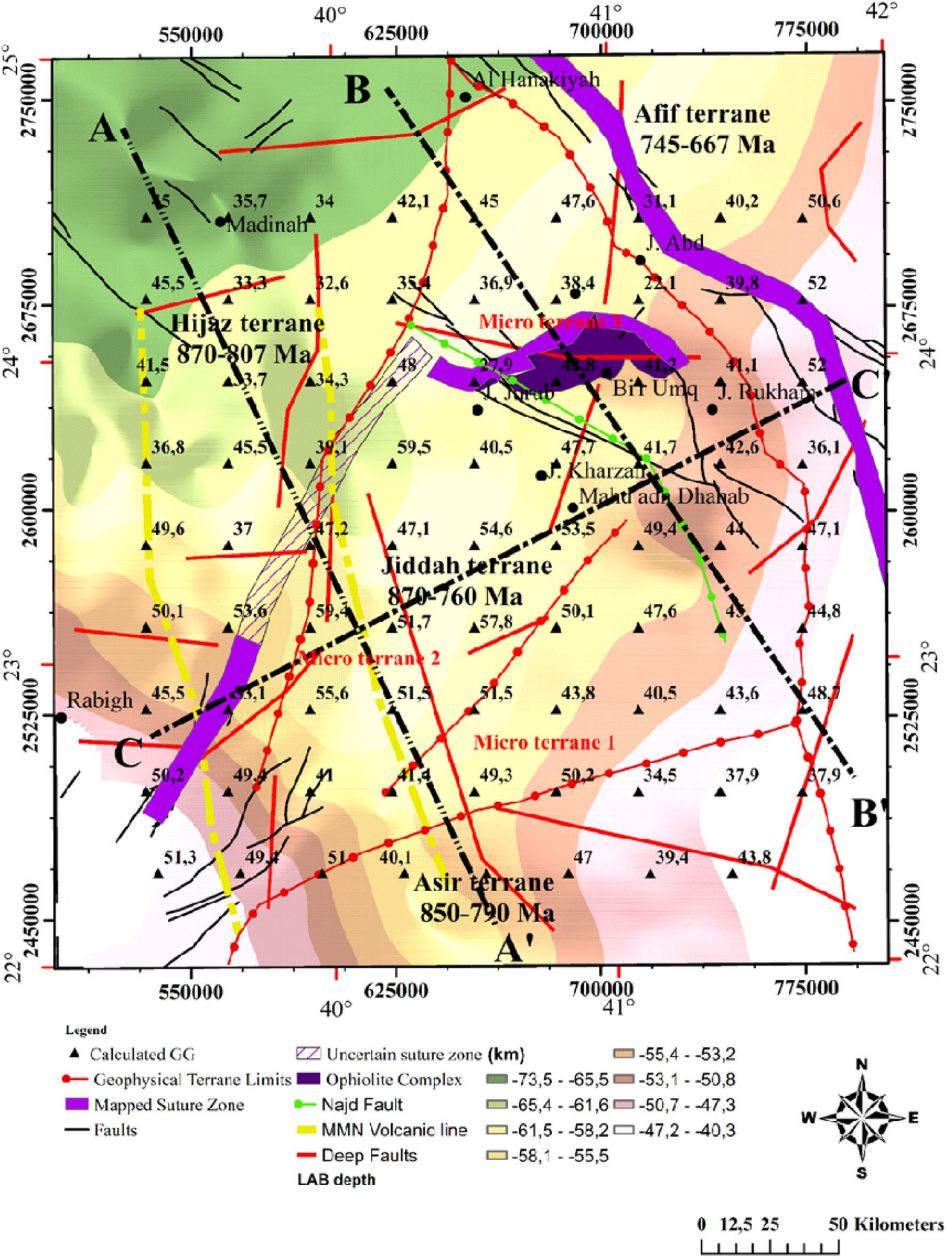
Lithosphere-asthenosphere boundary resulting from topography and geoid model, correlated with deep faults and terranes limits. Map generated using ArcGIS Pro (https://pro.arcgis.com/en/pro-app/latest/get-started/download-arcgis-pro.htm).

Generally, within the lithospheric mantle the LAB depth increases with thermo-tectonic age due to conductive cooling^132^. The obtained results (Fig. 13) show that the northwestern limit is thicker (62–73.5 km), while thinner lithospheric mantle was observed within the eastern border of the Red Sea rift (40–50 km) and to the southwestern limit of Afif terrane (40–61 km). Previous works estimated the LAB depth in the area to range between 70 and 105 km^131^. Estimated it to be 70–90 km depth^133^. Estimated the LAB using P- and S-waves at 40–100 km under the Arabian shield^126^. Suggested that effects of convection movements in association with extensional forces and mantle upwelling can generate an average thinning rate of ~ 7.5 km/Myr^134^. Used this rate to calculate the thinned lithosphere (with starting thickness of 160 km) underneath the Arabian shield while the estimated LAB stood at 50 km^126^. The obtained model agrees well with our results and attributes the age of the active rifting to 15–20 Myr. The actual LAB topography can be the result of two-stage rifting episode including extensional and erosional phases generated by asthenospheric heat flow.

### 2D thermal and seismic modeling

The input dataset used to build the model are available in the supplementary data section (supple. Figs. 4–6) and include surface topography, geoid model, free air, and Bouguer anomaly. The structure of the model was realized using the available information from this study (i.e., heat flow, basement depth, Moho model, LAB depth, and heat flow data). Observations from the three profiles (A–A′, B–B′, C–C′) suggests that the Afar plume transfer from the asthenosphere intrudes the lithospheric mantle with vertical structures having the same physical, geochemical and thermal properties as the asthenosphere. Also, the ophiolites in the suture zones were assumed to have the same origin across the entire area.

**Figure 14:**
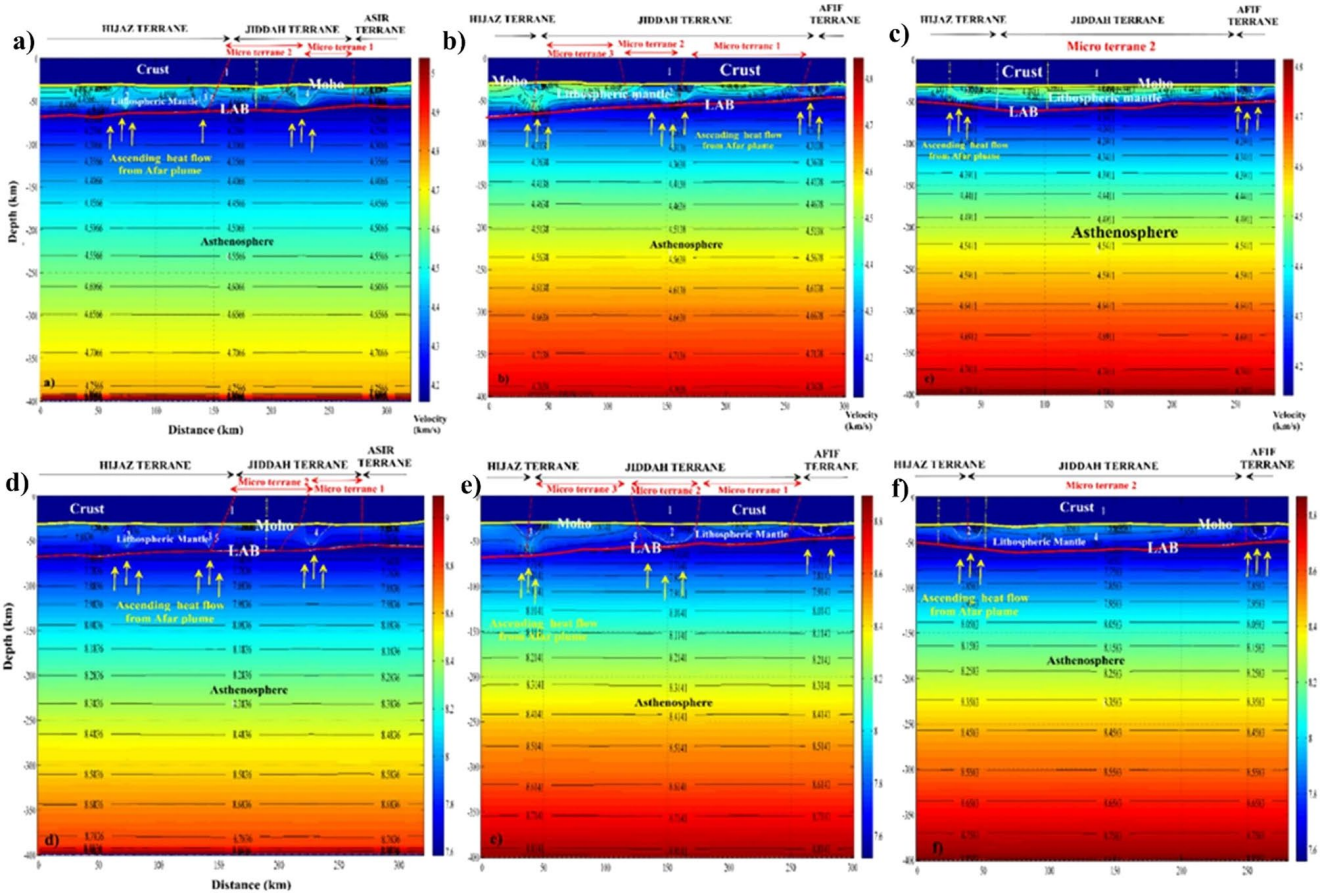
Distribution of S and P waves showing the crust, the mantle, and the asthenosphere along three profiles resulting from Litmod modeling: (**a**) S waves model along profile A–A′; (**b**) S waves model along profile B–B′; (**c**) S waves model along profile C–C′; (**d**) P waves model along profile A–A′; (**e**) P waves model along profile B–B′ and (**f**) P waves model along profile C–C′.

**Figure 15:**
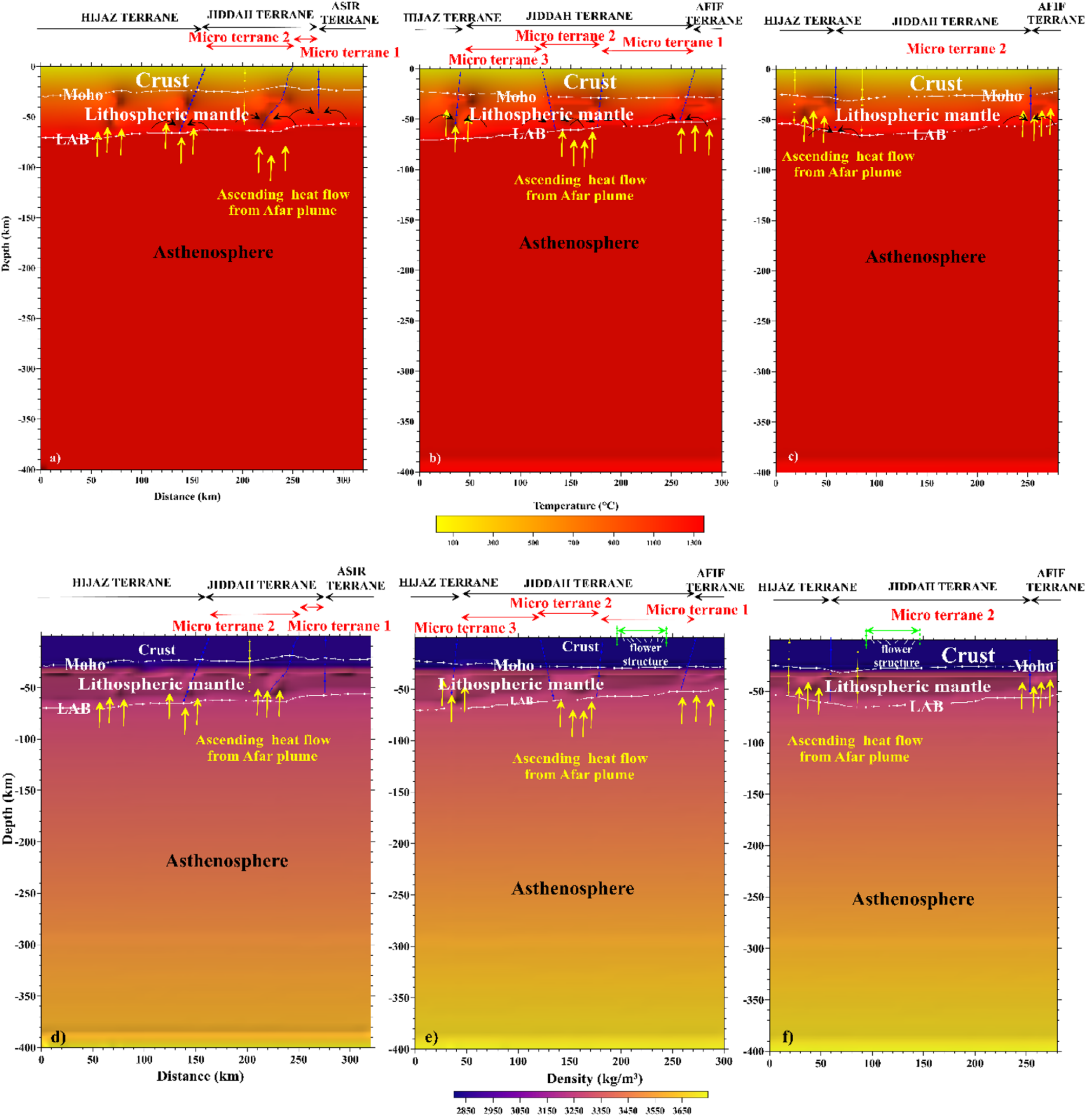
Distribution of Temperature and density showing the crust, the lithosphric mantle, and the asthenoshere along three profiles resulting from Litmod 2D modeling: (**a**) thermal model along profile A–A′; (**b**) thermal model along profile B–B′; (**c**) thermal model along profile C–C′; (**d**) density model along profile A–A′; (**e**) density model along profile B–B′; and (**f**) density model along profile C–C′.

Conversely to the LAB signature, thick crust was observed along the eastern limit of the MMN volcanic line. The P-waves velocity increases vertically from the crust towards the asthenosphere (~ 7.5 to 9.0 km/s). The limit of Hijaz-Jiddah is characterized by higher velocity, due to structural deformation generated during the collisional events (Fig. 14). The LAB limit decreases in depth from Hijaz towards the Afif Terrane. The P-wave velocity increases slowly to ~ 8.0 to 9.0 km/s below the 200 km depth. The S-wave model (Fig. 14) shows lateral homogeneity of crust structural, while the crust-lithospheric mantle contact show increase in the wave velocity to ~ 4.5 km/s. The uplifted bodies interrupted the lateral homogeneity of the lithospheric mantle, changing the speed of S-wave and generating large scale deformation attributed to activities associated with the deep root of the Harat volcanism. The contact between Jiddah micro-terrane is marked by significant deformation in the Moho and the LAB, indicative of probable asthenospheric intrusion. In Asir terrane, the LAB depth is estimated to vary from 50 to 55 km and changes in seismic wave velocities and differences in layer temperature were observed, indicative of deep cold root of the Cenozoic basalt. The thermal model (Fig. 15a) demonstrates that the temperature in the crust ranges between < 300 and 800 °C, while the temperature at the Moho ranges between 800 and 1000 °C. Within the lower crust, the suture zone is marked by significant disturbance and the temperature exceeds 1000 °C under the contact zones. The vertical extension of these structures appears to be colder (i.e., 900–1000 °C) than the lithospheric mantle (i.e., 1000–1200 °C).

The crustal density ranges between 2800 and 2900 kg/m^3^ (Fig. 15d). These values reflect the influence of the Cenozoic volcanism on the crust structure. The lithospheric mantle under the Harrat is characterized by density values of the range 3050–3270 kg/m^3^. The variation in crustal density is due to the contaminated plume from the asthenosphere with density values that range between 3270 and 3310 kg/m^3^. The asthenosphere density increases from the LAB to the model limit (i.e., 400 km depth) and reached 3550 kg/m^3^ at 300 km depth. Along the B–B′ transect, the crustal structure is thinner in the Hijaz terrane and the micro terrane 3 (Jiddah terrane). The suture zone is characterized by Moho that reaches 30 km and corresponds to the ophiolite complex uplift (Fig. 12). The structural variability observed within the crust-lithospheric mantle interface that can be attributed to plume upwelling, show P-wave velocity that vary from 7.8 to 7.9 km/s. The wave distribution within the continental crust reaches ~ 7.5 km/s, while it increases gradually to reach ~ 9.0 km/s at the asthenosphere (Fig. 14b). The thermal disturbance generated by plume upwelling is marked by increasing temperature rates at the crust-lithospheric mantle interface (Fig. 15b). The deformation between the Moho and LAB moderately influenced the temperature distribution model at the crust-lithospheric mantle interface along terrane limits^77^. Additionally, the model is slightly influenced by the structural events occasioned by ophiolite outcrops within the surface.

The density anomalies were encountered along the crust-lithospheric mantle interface generating sharp contrast in density of ~ 100 to 300 kg/m^3^. The crustal density is estimated to range between 2800 and 2950 kg/m^3^. These results agree with crustal density values from previous studies^1,20^. The near homogeneous crustal density observed is due to the similarity of the geologic materials along the Harrat provinces. Additionally, the estimated lithospheric mantle density range between 3150 and 3250 kg/m^3^ which agrees with the range of values (i.e., 3200–3260 kg/m^3^) reported^126^. Similarly, asthenosphere density was estimated to be 3120 kg/m^3^ by^126^, which is slightly below the value that was observed in this study from modeling results i.e., > 3350 kg/m^3^ (Fig. 15e). The C–C′ transect oriented SW-NE traverses the MMN volcanic line-Hijaz-Jiddah-Afif terranes along 280 km, demonstrating differential P- and S-waves propagation within the crust and mantle layers. The MMN volcanic line is the result of structural deformation, evident in the observed Moho and LAB discontinuities. These discontinuities are products of the mantellic convection process. The P- and S-wave velocities within the crust were observed to be the lowest reaching ~ 7.5 and 4.2 km/s respectively (Fig. 14). Terrane limits were marked by increasing wave speed due to changes in material; mainly of ophiolitic origin. The composition of the lithospheric mantle is dependent on the age of the overlying crust. The Archean and the Proterozoic cratons are the most depleted lithospheric mantle portions. The chemical composition of the lithospheric mantle in terms of Fe, Ca and Al contents had direct consequence on the geophysical properties. Lower content on Fe implies low densities and higher seismic velocities, while medium to higher Al content results in high seismic velocities. The uncertainties in the seismic velocities can be in the order of 10–15% depending on the validity of the geochemical compositions used as input and the amount of constraints applied in building the starting model. However, the presence of geochemical heterogeneities introduced by the Afar plume and the local melting process can also result in decrease in the deep crustal seismic velocities.

The thermal and density models illustrate the same vertical increasing trends as the seismic models. The crust-mantle temperature increases from < 300 to 800 °C at the crustal surface to ~ 800 to 1300 °C within the Lithospheric mantle and > 1300 °C below the asthenosphere. Also, crustal density was observed to range between 2800 and 2950 kg/m^3^ and increases to ~ 3000 and 3250 kg/m^3^ within the lithospheric mantle, and > 3350 kg/m^3^ below the asthenosphere (Fig. 15c, f). Table 3 summarizes the results of comparative studies in the region using different geophysical approaches. The use of geothermal and geophysical modeling setup provides the structural dynamic trends of the Western Arabian Shield, and gives realistic interpretations of the conceptualized origin of geothermal anomalies.

**Table 3:** Comparative studies showing: crust depth, LAB depth, velocities of P and S waves, temperatures and densities in the western Arabian Shield.

Layer	Depth (km)	P-wave velocity (km/s)	S-wave velocity (km/s)	Density (kg/m^3^)	Temperature (°C)	Source
Crust (including sediments)	25–30	–	3.8–3.9	–	–	Pasyanos and Walter^135^
Lithospheric mantle	–	–	4.25–4.35	–	–	
Crust (including sediments)	30–40	–	–	2800–2850	–	LithRef18 (Afonso et al.^136^)
Lithospheric mantle	60–100	–	–	3240–3280	–	
Asthenospheric mantle	–	–	–	3440–3455	–	
Crust (including sediments)	28.6–32.6	7.5–7.8	4.2–4.3	2800–2950	< 300–800	This study
Lithospheric mantle	40.3–73.5	7.8–7.9	4.3–4.5	3000–3350	800–1150	
Asthenospheric mantle	–	7.6–9.0	4.2–5.0	3350–3700	> 1200	

### Correlation of gravity, aeromagnetic, heat flow and topography

**Figure 16:**
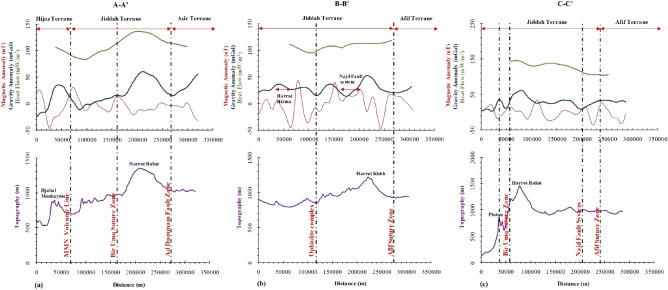
Correlation of gravity anomaly, aeromagnetic anomaly, heat flow and topography. (**a**) signatures of different tectono-thermal events along the cross-section, A–A′, (**b**) signatures of different tectono-thermal events along the cross-section, B–B′ and (**c**) signatures of different tectono-thermal events along the cross section, C–C′.

The terrane limits (Fig. 16a) are marked by three major tectono-thermal events, i.e. the MMM volcanic line, Bi’r Umq suture zone and the Ad Dammam fault zone. The Hijaz Terrane shows high-amplitude aeromagnetic anomaly (i.e., short wavelength anomaly), negative gravity anomaly and high heat flow associated with near-surface volcanics. Magnetic highs were due to the ascension of post-tectonic granitoids. High magnetic signatures due to magnetized rocks, were also observed within the new arc-arc suture zone between Hijaz-Jiddah terranes and the Bi’r Umq suture zone. The limits of Hijaz and Jiddah display alternating sequences of positive and negative gravity and aeromagnetic signatures, respectively that are indicative of collisional events (Fig. 16a). However, towards the Bir Umq suture zone, both gravity and magnetic anomalies were positive. Beyond the suture, the gravity anomaly and heat flow increased towards Harrat Rabat. The strong positive gravity anomaly, negative magnetic anomaly and high heat flow correlates with the high relief of Harrat Rabat and Kishb. These signatures are associated with dense basaltic rocks (i.e., the Cenozoic basalt) resulting from the activities of the Afar plume and melts within the lithosphere-asthenosphere boundary (Fig. 16a,b). Generally, the high temperature due to the plume flow considerably influenced the magnetic field intensity within the Harrat. Moderate magnetic and low gravity signatures within the Ad Dammam fault zone that separates the Jiddah and Asir tectonostratigraphic terranes was observed. The magnetic lows within the Asir terrane are products of subduction tectonics and graben structures. In Fig. 16b, the Ophiolite complex led to suture zones and high strain shear zones. The alternate high and low gravity and magnetic signatures are due to the variations in ophiolite geochemistry and thermodynamic characteristics during the extensional and collisional phases. The Najd fault system is marked with high magnetics and low gravity signatures (Fig. 16b,c). The variations can be attributed to the similarity in timing of extensional events and the source of melting materials during ascendant flow. The Afif suture zone is marked by similar gravity and magnetic signature as the Ophiolite complex reflecting the same tectono-structural condition (Fig. 16b,c).

## Discussion

The use of multidisciplinary approach that integrates geophysical and geological data provides new insights to the relationship between crustal structures and the distribution of thermal properties within the Western Arabian Shield. Potential field data demonstrates the consistency between the geological terrane boundaries and the thermal regime that defined the WAS. Sutures between the Tonian terranes (i.e., 1000–850 Ma) and Cryogenian (i.e., 850–650 Ma) have been postulated since mid-1970’s^137^, isotopic and geochemical arguments based on assessment of mafic and ultramafic rocks yield outcomes that are uncertain to some extent^138^. Recognizing the characteristics of this area in terms of location and age is crucial and provides insight into the crustal accretion processes within the Arabian-Nubian shield. Studies from^139^, reported the non-availability of geophysical data in the region to interpret the 3D structures that exist within Bi’r Umq suture and suggest a subvertical lithospheric-scale structure. The compilation of geophysical interpretation suggests new limits of terranes in the WAS, which corresponds to high magnetic signatures. Similar high magnetic intensities have been reported within the limits of the West African Craton (WAC) in the Algerian Sahara^2^. To the eastern limit of the Red Sea, Bi’r Umq suture extends towards Nakasib suture and can be considered as a structural continuity. The Nakasib-Bi’r Umq shear zone can be interpreted as an arc-arc suture^139^, and show evidence of progressive folding, refolding of sheath folds, variable thrusting and non-coaxial deformation shears that are typical characteristics of developed shear zone during oblique transpressional stress regime.

Geophysical evidence of negative flower structure indicates the transtensional rotational movement along the Afif suture. More so, similar structures have been identified in the southern limit of Nakasib suture in Egypt^140^. Also, the origin of the Harrats Cenozoic basalts, based on the observed structural trends within the area, was linked to the Afar plume migration, the extensional tectonics and rifting beneath the Red Sea. Additionally, these processes have given rise to Moho distension and crustal thinning.

In order to interpret the origin of the geothermal heat flux, the regional geodynamic model^126^ of the Arabian-Nubian Province was used. It suggests that the mantle temperature may not have had time to equilibrate on the surface. This disequilibrium proves that the geodynamic processes that are currently shaping the Red Sea oceanic lithosphere were initiated between the last 15–20 Ma and probably reflects a second stage of rifting. The convection upwelling currently leads to some degree of partial melts. Consequently, this produces high velocity contrast across the lithosphere-asthenosphere boundary. Reports of^141^ suggests that the thermal equilibrium of the lithosphere has been modified beneath the Arabian Peninsula and that there exists significant abrupt alteration in the configuration of lithospheric structure within the Arabian plate. The aforementioned conditions of thermo-structural behavior of the lithosphere agree with the observations of the origin of heat flow and Moho deformation within the Western Arabian Peninsula. Generally, the emitted thermal flux density decays with age and tectono-structural stability. The suture zone flanked by the West African Craton and the Pan-African domain in Western Algerian Sahara can be an excellent example that describes the phenomenon.

**Figure 17:**
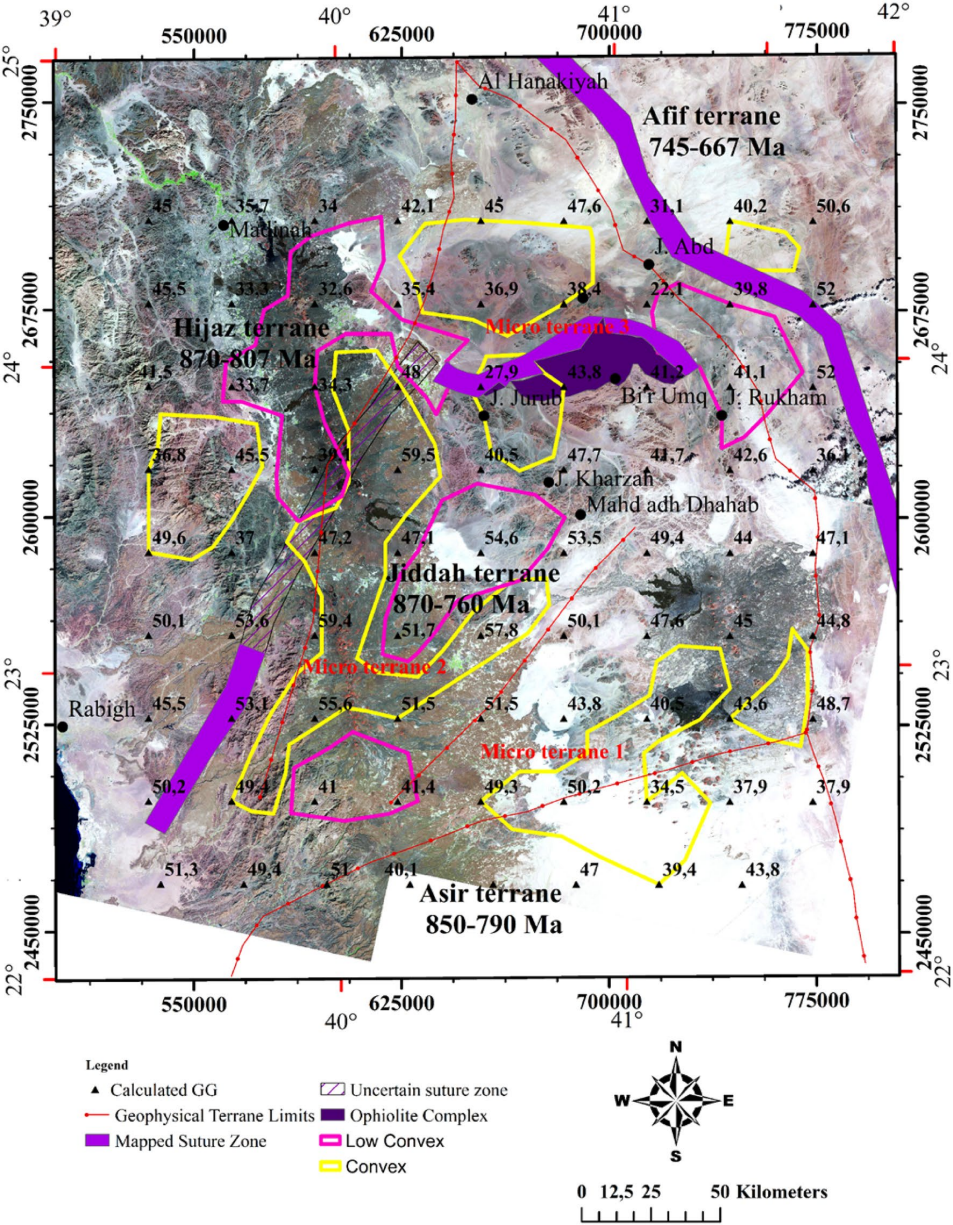
Spatial distribution of potential geothermal exploration zones within the Western Arabian Shield. Pink and yellow lines respectively represent low convex reservoir and convex reservoir types, black rectangle represents the limits of negative flower structure, black arrows represent distension/compression forces. Map generated using ArcGIS Pro (https://pro.arcgis.com/en/pro-app/latest/get-started/download-arcgis-pro.htm).

The classification of geothermal reservoirs was based on evaluation of major factors that can adequately distinguish geothermal anomalies within the region. In the WAS, geothermal gradient which is a dominant factor was employed during the classification. Hence, three dominant classes^142^, were identified. However, the convex and low convex types constitute the economic exploration targets (Fig. 17). The convex geothermal reservoirs are usually associated with uplift areas where depth of burial of the basement range from ~ 2000 to 4500 m and amplitudes of vertical faults range between 1000 and 5000 m^142^. Within the area, geothermal values of convex gradient class range from ~ 22 °C to 42 °C/km. The nature of rock formation and the Red Sea lithospheric extensional tectonics, tend to diffuse heat from deep sources. Consequently, this process leads to increased thermal perturbation resulting in large geothermal heat flux within the area. The Furayh eastern group, Ghamr group and Amudan formation are representatives of the convex type geothermal reservoirs within the Paleozoic aquifer and possess good potentials for development of geothermal energy. The low convex class of geothermal reservoirs commonly occur where basement depths range between ~ 4500 and 6000 m and major extensional fault amplitudes within these regions are between 2500 and 6500 m. Depending on the fault network extensions and the local geodynamic systems, the geothermal gradient of the low convex type within the Paleozoic aquifer range between 22 and 44 °C/km, hence, can serve as a geothermal reservoir target. As explained previously the validation criteria are based on the depths of faults, basement depth and the values of the geothermal gradient^142^. The deep burial type (44 and 59 °C/km) corresponds to specific structural trends within the study area. The majority of deep buried reservoirs are oriented in the NE–SW direction, dominant in secondary-tertiary sedimentation (> 6000 m in thickness).

**Figure 18:**
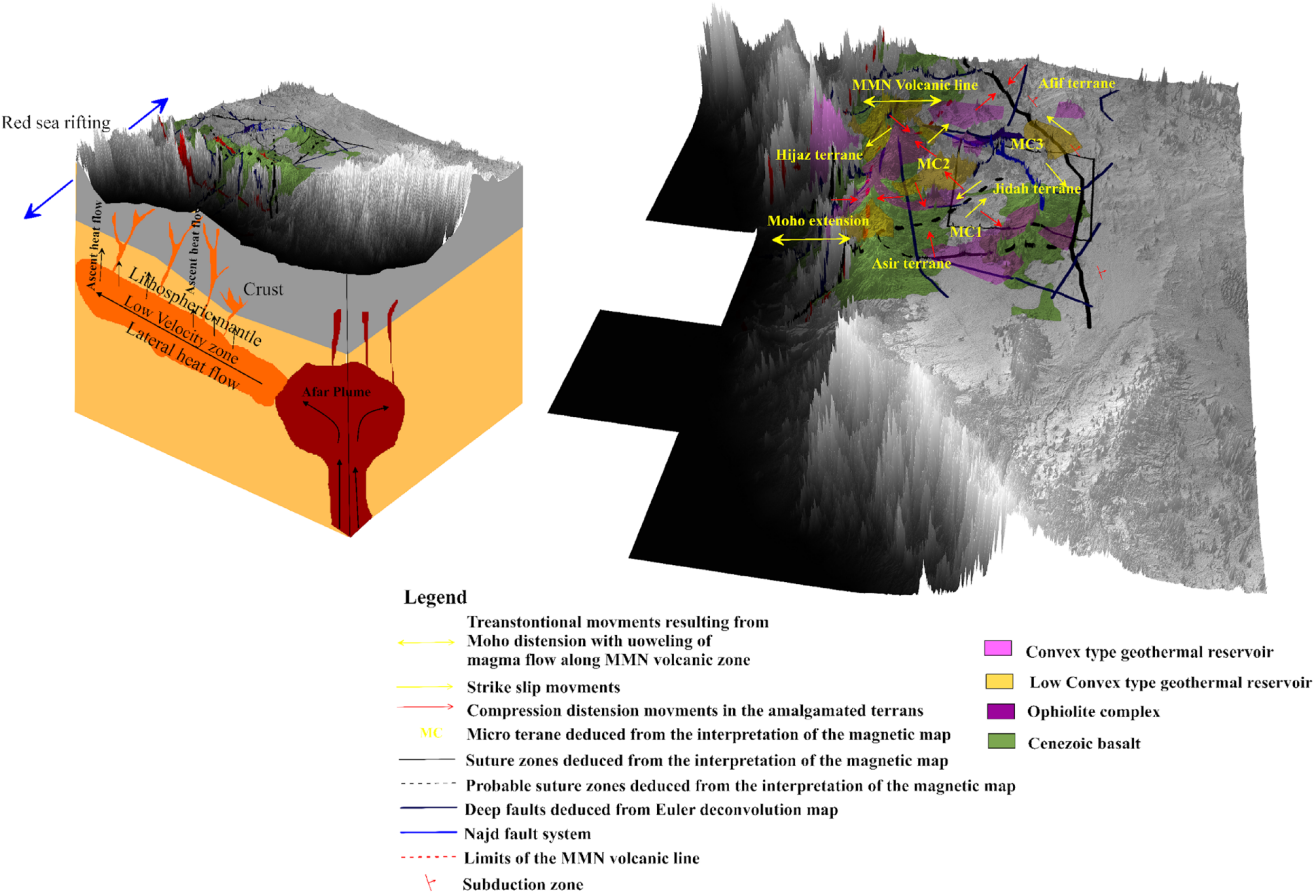
3D block diagram showing heat flow transfer mechanism in the Arabian shield Map generated using Golden Software Surfer V23 (https://support.goldensoftware.com/hc/en-us/articles/226806288-Download-my-software-online).

In order to provide answers to the knowledge gap concerning the origin of heat flow in the Arabian shield, it is important to locate the source magma in the Harrats. Some earlier studies attributed the origin of magma in the WAS to partial melting of incomplete enriched elements in the mantle^143,144^. However, geochemical research disagreed with this conjecture and suggested that the magma in Saudi Arabia, Syria and Jordan originated from beneath the Arabian plate, i.e., lithospheric mantle^145^. Binary mixing linked to lithospheric-asthenospheric melts was adjudged to be responsible for the magma composition around Jordan^146^. Provided a contrary view concerning the Afar plume as the source of magma in the area and justified it by the flow speed of the Red Sea rift (~ 30 mm/years) covering the western Arabia^133^. Focused research on the Harrat zone (i.e., central and northern Arabian shield) attributed its origin to extension of the lithosphere produced by the Red Sea rifting^147,148^. This geophysical study proposes that the northward migration of magma flow from the Afar plume drives local mantle melts beneath Western Arabia, thereby providing the pressure field required for magma mixing and ascent^149–154^. The ascendant magma flow provides the heating source of geothermal reservoirs within the WAS. Studies of^155^ showed that the Afar plume located beneath southwestern Arabia, flows radially away from its origin. Coincidentally, one of its axes which flows in the direction north, flows beneath Western Arabia, while the other axes are eastwards beneath the Gulf of Aden and southwesterly beneath Ethiopia. The mixing origin of magma due to the proximity of the Afar plume and the local melts beneath the WAS may have been influenced by the ancient Pan-African activities and/or earlier phases of rifting within the Red Sea, resulting in crustal thinning and providing pathways for ascendant magma flow along the MMN volcanic line leading to fractional crystallization of magma (Fig. 18).

## Conclusion

The analysis of structural observations combined with gravity and magnetic data provides new insights and interpretations to the crustal architecture in the WAS. Several observations were deduced from the geophysical anomalies of deep and shallow sources. The residual gravity anomaly corresponds to deep structures whereas the residual magnetics represent shallow lying sources and boundaries of the tectonic units. Comparative assessment of edge enhancement techniques illustrates the effectiveness of both LTHG and ILTHG filters to detect structural trends due to sutures and shear zones tectonic activities.

Negative flower structure has been identified between Bi’r Umq and Afif sutures provides evidence of transtensional divergence typical to wrench fault zones. This research has not only provided new sets of micro-sutures required to reconstruct the geodynamic-structural interpretation of the crust within the WAS, but has also provided new evidence of the origin of the geodynamic characteristics and thermal anomalies, represented by a conceptual model. The classification of the geothermal resources is controlled by the basement depth, the geothermal gradient and the behavior of the tectono-structural elements. Hence, the convex, low convex and deep buried geothermal reservoirs were identified. Based on economic considerations, the convex and low convex types were preferred. This paper exposes original results that were developed using a multi-approach concept. The presented model satisfies various geophysical datasets and highlights the tectono-thermal trends and provides a consistent scheme for characterizing the crust-mantle structures. The thermal structure provides good correlation with the magnetic, gravity and heat flow modeling results. The estimation of Moho and LAB discontinuities provides significant insights to the supposed structure of the Red Sea rifting and the Afar plume. Hence, contributes to improving the hypothesis on the source of the thermal heat flow in the WAS.

## Supplementary Information

Supplementary Figures.

## Data Availability

The datasets generated and/or analyzed during the current study are available in the [Science Data Bank] repository, [https://www.scidb.cn/s/2QvyAb].
