# Supplementary material for: Lithospheric structural dynamics and geothermal modeling of the Western Arabian Shield
Source: Sci Rep. 2023 Jul 20;13:11764. doi: 10.1038/s41598-023-38321-4 (PMC10359420; doi:10.1038/s41598-023-38321-4)
Supplement: Supplementary file 1 — Supplementary Figures. [file 41598_2023_38321_MOESM1_ESM.docx]

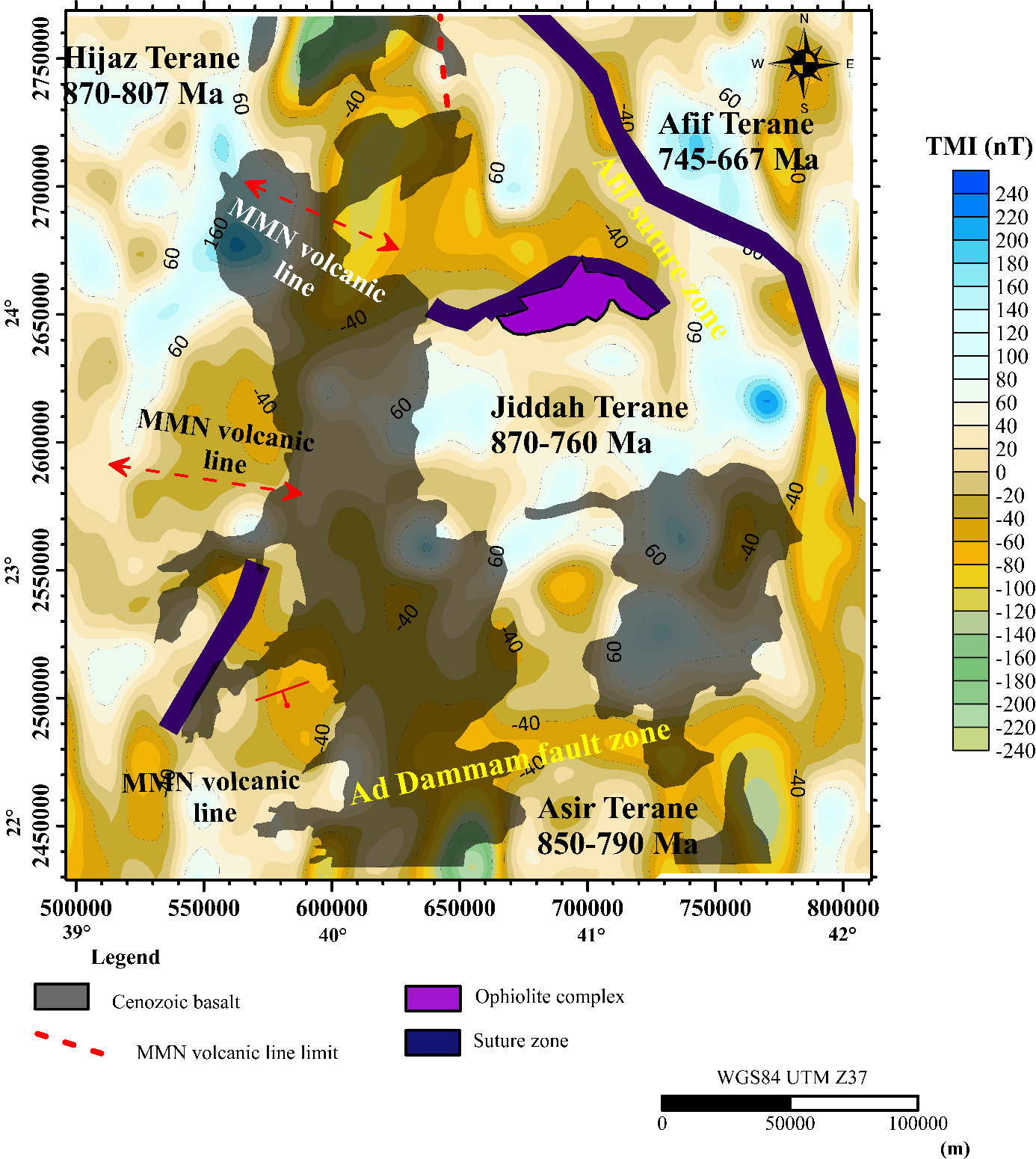


**Supple. fig 1 :** Total magnetic intensity map of the study area derived from EMAG2 magnetic model


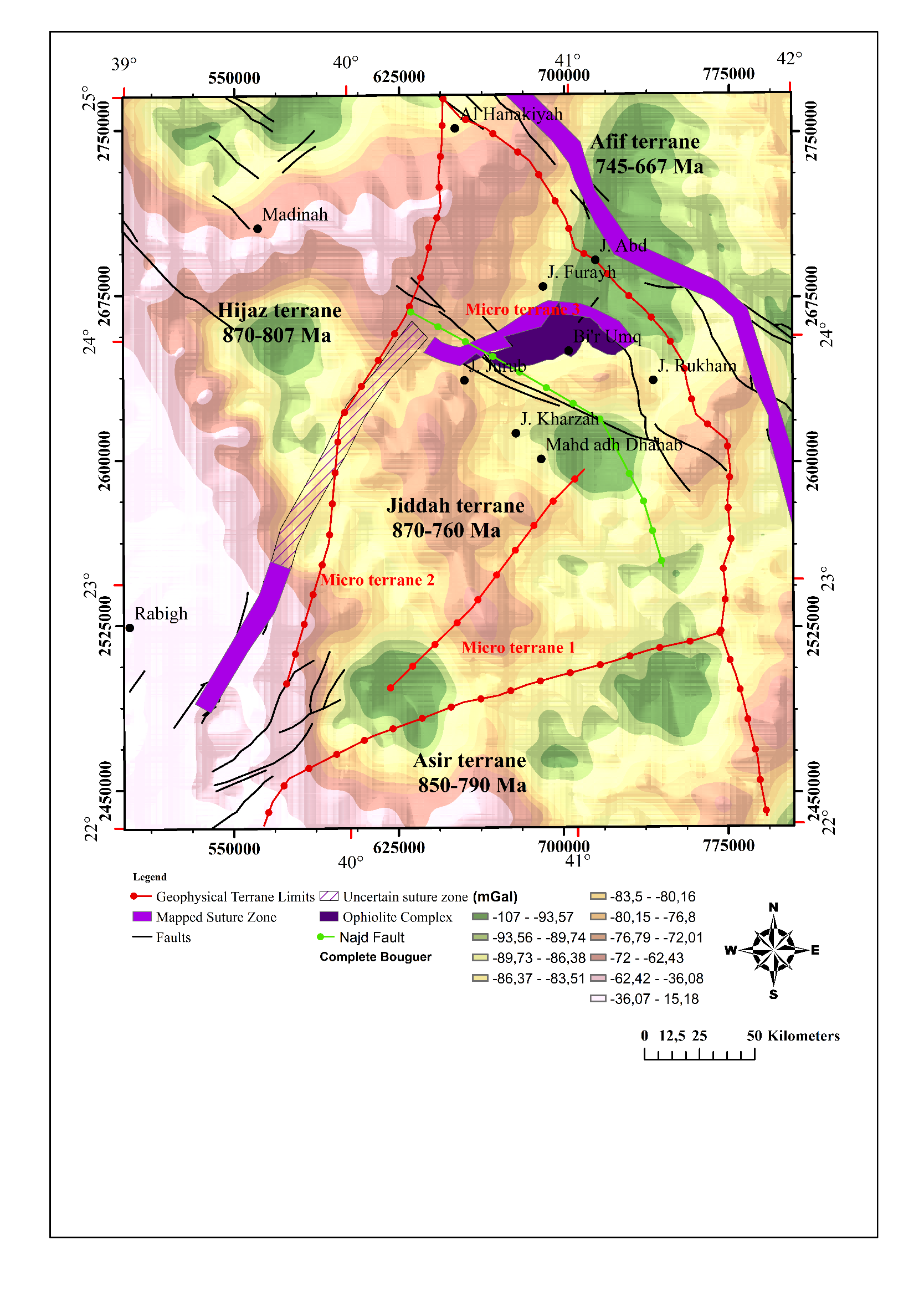


**Supple. fig 2:** complete Bouguer anomaly map of the study area derived from EIGN6C4 gravity model


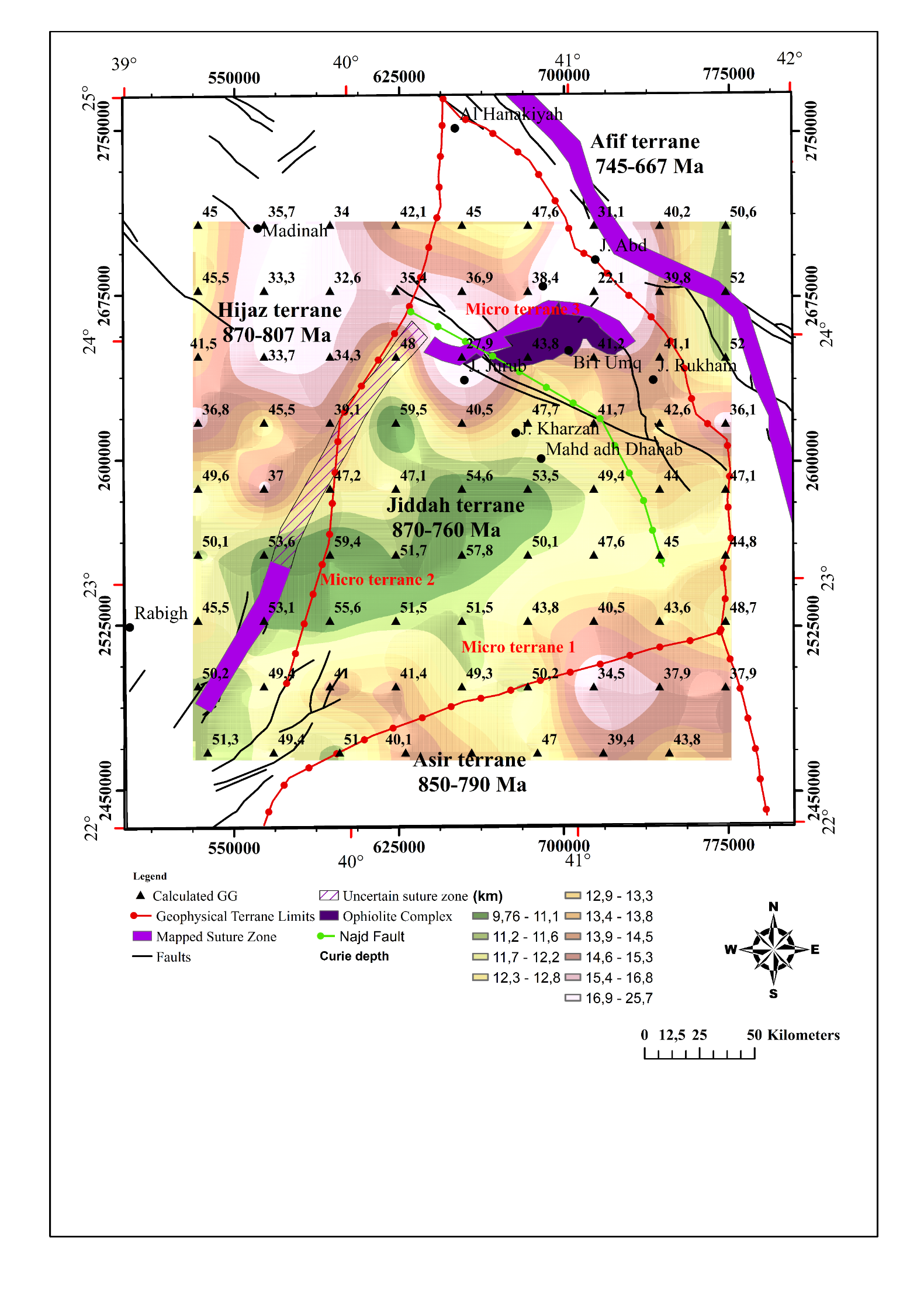


**Supple. fig 3 :** curie depth map of the study area derived from centroid method


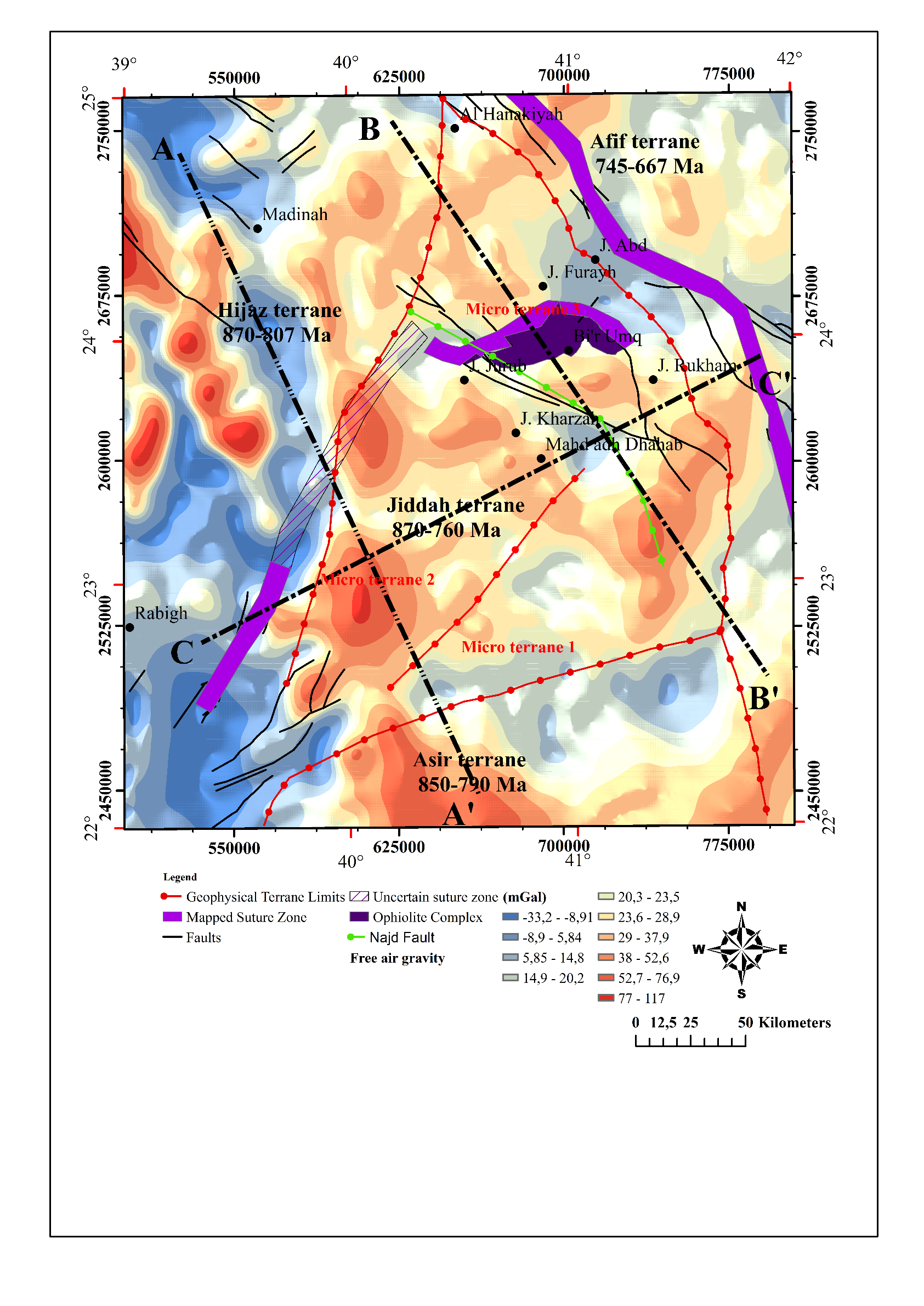


**Supple. fig 4 :** free air gravity map of the study area derived from BGI gravity database


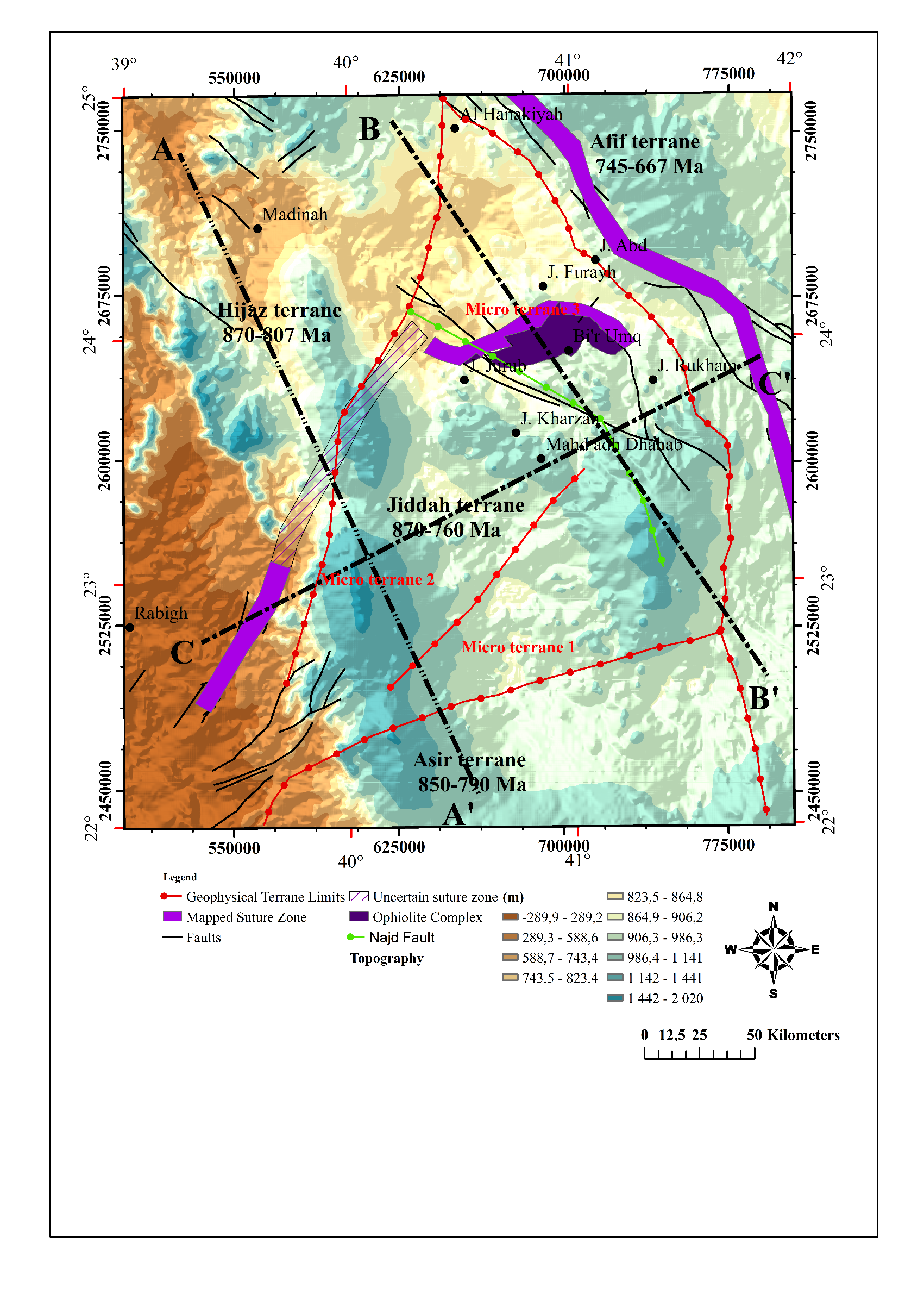


**Supple. fig 5 :** Topography map of the study area derived from BGI gravity database


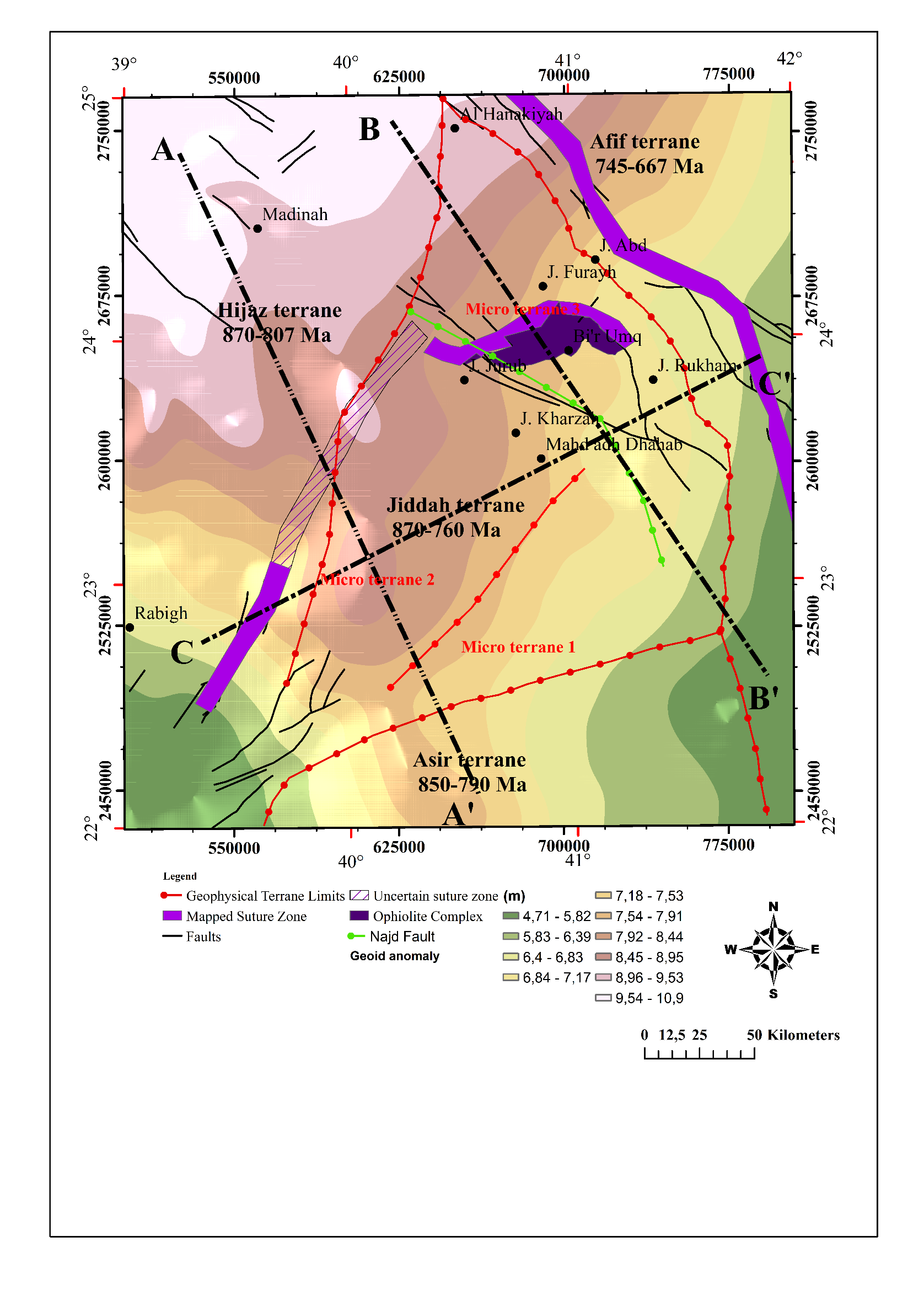


**Supple. fig 6 :** Geoid anomaly map of the study area derived from EICGN6C4 database
